# Enhancing musculoskeletal examination skills through near-peer teaching: student outcomes and perspectives

**DOI:** 10.1186/s12909-026-09235-2

**Published:** 2026-04-18

**Authors:** Kevin Qian, Bunmi Malau-Aduli, Keran Sundaraj

**Affiliations:** 1 The University of Newcastle, Callaghan, NSW Australia; 2 Hornsby Ku-Ring-Gai Hospital (Northern Sydney Local Health District), Hornsby, NSW Australia; 3 North Sydney Orthopaedic Research Group, Wollstonecraft, NSW Australia

**Keywords:** Near-peer teaching, Musculoskeletal examination, OSCE, Medical education, Clinical skills, Confidence

## Abstract

**Background:**

Musculoskeletal (MSK) conditions are highly prevalent and represent a major burden in Australia and worldwide, yet undergraduate musculoskeletal education remains underrepresented in medical curricula. Near-peer teaching (NPT) has emerged as an effective adjunct to standard teaching, particularly in developing clinical examination skills.

**Objective:**

To examine the effect of a near-peer-led, OSCE-style tutorial on medical student performance of the upper limb musculoskeletal examination, and to explore students’ perceptions of its clarity, relevance, and educational value.

**Methods:**

A quasi-experimental pre–post study was conducted with 23 second year-year medical students in a problem-based learning program. Participants completed a pre-test OSCE, attended a near-peer-led tutorial, and undertook a post-test OSCE three days later. Assessments were scored using a 15-item rubric with percentage conversion. A post-hoc survey comprising Likert-scale and open-ended questions explored student perceptions. Quantitative data were analysed using paired statistical tests, while qualitative responses underwent content analysis.

**Results:**

Improvements were observed in 14 of 15 OSCE items, with statistically significant gains in shoulder and elbow palpation, shoulder active and passive range of motion, patient comfort, and examination organisation (*p* < 0.05). The overall average performance improved from 63.8 ± 15.8% pre-test to 76.3 ± 10.5% post-test (*p* = 0.01). Survey responses (*n* = 17) indicated that all students perceived the tutorial as superior to lectures, textbooks, or videos, with 59% rating it *extremely useful* for clinical practice. Thematic analysis revealed strengths in logical organisation, integration of anatomy and pathology, demonstration, and opportunities for hands-on practice. Most students reported substantial gains in confidence and understanding. Findings support the utility of NPT in musculoskeletal education, with improvements potentially explained by cognitive and social congruence, experiential learning, and self-efficacy. While a single session significantly enhanced competence and confidence in many components of the physical examination, skills such as special testing requires repeated practice and longer-term follow up to observe similar gains.

**Conclusion:**

A single near-peer-led, OSCE-style tutorial supports improvement in medical students’ objective performance and self-reported confidence in conducting the upper limb musculoskeletal examination. It is a beneficial adjunct to traditional musculoskeletal curricula for both learners and tutors. Broader, longitudinal research is needed to evaluate long-term knowledge retention and generalisability.

**Supplementary Information:**

The online version contains supplementary material available at 10.1186/s12909-026-09235-2.

## Introduction

Musculoskeletal (MSK) complaints are among the most common reasons for patients to seek medical care, yet evidence suggests that many physicians and trainees are underprepared to manage such conditions [1]. Globally, MSK disorders are a leading cause of disability: they account for 25% of non-fatal disease burden [2] and in 2023, contributed 13% of the total disability-adjusted life years (DALYs), ranking as the third most burdensome disease group worldwide [3]. In Australia, one in five general practice consultations during 2015–2016 involved a musculoskeletal complaint, representing an estimated 4.1 million encounters [2, 4]. These statistics highlight the need for strong musculoskeletal education during undergraduate medical training.

In recognition of this, musculoskeletal conditions were designated a National Health Priority Area in Australia in 2002, prompting the development of the Australian Core Competencies in Musculoskeletal Basic and Clinical Science project in 2005. This initiative reviewed musculoskeletal education across 17 medical schools, revealing major gaps. Faculty reported that insufficient time was allocated for students to achieve core musculoskeletal knowledge, while the Australian Medical Students’ Association (AMSA) found that students experienced “insufficient or conflicting training” in basic examination skills and lacked confidence in managing routine musculoskeletal cases [5]. A subsequent survey of 44 medical schools in 10 Asia–Pacific countries reported that only 2% of total teaching hours were devoted to musculoskeletal and rheumatology content [6], a figure that is consistent with findings from the United States (US), United Kingdom (UK), and Canada [7–9]. These trends underscore an urgent need to strengthen musculoskeletal teaching and ensure graduates are competent in performing musculoskeletal examinations [10].

At the same time, contemporary medical curricula have increasingly shifted from traditional didactic lectures to problem-based learning (PBL) and other student-centred approaches. PBL emphasises small-group, case-based learning facilitated by tutors, which enhances student interaction, critical thinking and engagement [11]. Many medical schools already leverage peer-assisted learning in PBL settings, where students learn collaboratively through guided discussions and feedback [12]. While valuable for conceptual knowledge, PBL alone is insufficient for mastering hands-on physical examinations such as the upper limb musculoskeletal exam, which require structured practice and feedback. To address these gaps, innovative strategies such as near-peer teaching (NPT) have emerged as effective adjuncts to faculty-led instruction [13].

NPT is defined as teaching delivered by a trainee at least one year senior to the learner [14, 15]. In the past decade, Australian medical schools have formally integrated NPT into their curricula [15]. Evidence shows that NPT improves both clinical skills and OSCE performance when used alongside standard teaching [16, 17]. Benefits extend to both learners and facilitators: students gain competence, confidence, and relatable guidance, while tutors consolidate their own knowledge and develop transferable skills in communication, leadership, and teamwork [15, 18]. NPT also fosters a culture of learning and teaching, shaping professional identity and preparing future clinicians for expected roles in medical education [19]. Theoretical frameworks of cognitive and social congruence can help explain NPT’s effectiveness. Cognitive congruence refers to the tutor’s ability to pitch teaching at an appropriate level due to their recent experience of learning the same material [20, 21]. Social congruence reflects the rapport and trust that develops between peers, reducing anxiety and encouraging honest disclosure of uncertainties [17–24]. Together, these factors create a safe, motivating environment that enhances student satisfaction, confidence, and performance [13, 15, 25].

Despite these benefits, some challenges exist. Near-peer facilitators have less depth of knowledge than faculty [26], and without adequate preparation, may resort to extrapolation or provide incomplete information [27, 28]. Content can also vary depending on the tutor’s priorities, risking selective learning [15]. Despite these limitations, a growing body of evidence supports NPT in musculoskeletal education. For example, a recent peer-led orthopaedic teaching programme in the UK demonstrated a significant increase in student and tutor confidence in musculoskeletal examination skills and orthopaedic knowledge as assessed through a multiple-choice test [29]. This is supported by another peer-led module achieved a 93% Objective Structured Clinical Examination (OSCE) pass rate compared to 67% in students exposed only to the standard curriculum [30].

Given the centrality of the OSCE in medical education, NPT has been particularly effective when delivered through OSCE-style teaching. OSCEs, introduced in the 1970s, provide structured, standardised assessment of clinical competence across history taking, examination, communication, and management [16, 31–33]. They are now widely regarded as both a reliable summative assessment and a valuable formative learning tool. Yet OSCEs are also a source of considerable anxiety for medical students, who often feel unprepared due to limited opportunities for rehearsal under exam-like conditions [34]. Near-peer facilitated mock OSCEs have been shown to reduce stress, increase confidence, and improve performance [35, 36]. A recent mixed-methods study at the University of Adelaide reported that for every 1% increase in student-led OSCE performance, there was a 0.49% increase in summative OSCE marks, along with reduced stress and improved recognition of learning gaps [16].

Despite these promising outcomes, gaps remain. Much of the literature focuses on longitudinal peer-led programmes, leaving limited evidence on the impact of single, focused near-peer tutorials, particularly in pre-clinical musculoskeletal training. Furthermore, few studies explore post-hoc evaluations of single, focused near-peer-led OSCE-style tutorial, particularly in pre-clinical musculoskeletal education which could provide valuable insights into their perceived effectiveness, limitations, and role in fostering confidence and competence. By evaluating both objective performance outcomes and students’ perceptions, this study addresses an important gap and provides practical insights for integrating peer-led strategies into problem-based medical curricula.

### Study objectives

This study aimed to evaluate the impact of a single, near-peer-led, OSCE-style tutorial on the clinical performance and perceived confidence of pre-clinical medical students in conducting the upper limb musculoskeletal examination. Specifically, the study pursued the following objectives:

1. To assess the effectiveness of a near-peer teaching intervention in improving students’ objective performance on a structured clinical examination (OSCE) of the upper limb musculoskeletal assessment.
2. To explore students’ perceptions of the tutorial’s educational value, clarity, structure, and practical relevance, as well as its impact on their self-reported confidence and preparedness for clinical practice.

## Methods

### Study context and setting

This project was conducted over a 1-week period. The pre-test was conducted 1-week after the participants had received their university facilitated teaching on the upper limb musculoskeletal examination. The near peer led tutorial was conducted on the same day, after the pre-test. The post-test was conducted 3 days after the tutorial. The scheduling of the pre-test, intervention and post-test was predominantly determined by student availability around their formal teaching and timetable. The post-intervention survey (Appendix 1) was delivered via email to the participants after the tutorial. Student responses to this survey were gathered over the following 2 weeks. This survey was primarily developed for the purpose of evaluating student perspectives in this study.

The University of Newcastle Joint Medical Program’s (UON JMP) Peer Assisted Study Sessions (PASS) and the Near Peer Medical Teaching (NPMT) initiative by junior medical officers in the Central Coast Local Health District are prominent examples of successful, locally delivered NPT [37, 38].

This project involved the delivery of one near peer led tutorial to the Year 2 UON JMP students in Semester 1 of 2025, covering the upper limb musculoskeletal examination as an adjunct to the faculty led clinical teaching sessions. This tutorial was facilitated by a final clinical year UON JMP student in a classroom based face-to-face fashion at the Central Coast Clinical School (CCCS) in Gosford NSW.

The tutorial provided in this study (Appendix 2) covered the key aspects of the upper limb musculoskeletal examination that are assessed in the UON JMP upper limb musculoskeletal examination marking rubric (Appendix 3), with the addition of clinically relevant anatomy and holistic aspects of the examination that were not directly assessed in the rubric, such as the hand and wrist examination. The tutorial and presentation were peer-reviewed and approved by the last-named author (KS), who was the clinical supervisor for this project.

### Study design

The study employed quasi-experimental, mixed-methods, pre-test and post-test design to evaluate the impacts of a near-peer-led tutorial on student performance in the upper limb musculoskeletal examination and perspectives relating to the value and efficacy of NPT in musculoskeletal teaching. Both the pre-test and post-test were designed to replicate the current UON JMP Year 2 clinical examination OSCEs.

### Participants and assessors

Thirty-six second-year medical students were enrolled in the UON JMP based at the CCCS in Semester 1 of 2025. All students who were invited to participate in this study participated in a face-to-face meeting where the contents, objectives and potential educational benefit of the study was discussed. All students were given a physical document outlining how the data collected from this study would be used, and the contents and objectives of this study. Over 4-weeks, three separate reminder emails were sent to the entire cohort. Ultimately, 23 out of the 36 students opted in as participants.

Three assessors were selected to examine the students during the pre-test and post-test respectively to meet time demands. All 3 assessors are currently registered and practising physiotherapists in private settings focused on the assessment and treatment of musculoskeletal disorders. Two of the assessors were final-year medical students enrolled in the UON JMP, and the other was a penultimate medical student enrolled in the Doctor of Medicine program at Western Sydney University. Assessors were recruited based on expressions of interest on a voluntary basis, and familiarity with the upper limb musculoskeletal examination from both a curricular and clinical perspective. No formal interview or meritocratic assessment was used to recruit assessors.

### Data collection

#### OSCE Assessments

Each participant completed an 8-minute Objective Structured Clinical Examination (OSCE) station, involving a clinically unremarkable patient in a simulated setting. Performance was assessed by trained examiners using the standard JMP upper limb musculoskeletal examination marking rubric. To ensure consistency, the principal investigator (KQ) conducted a 30-minute calibration session with all assessors prior to the study. This briefing familiarised examiners with the rubric and standardised the interpretation of each assessment item.

The rubric comprised 15 individual marking items, each scored on a five-point scale: *unsatisfactory (U)*,* marginally unsatisfactory (MU)*,* marginally satisfactory (MS)*,* clearly satisfactory (CS)*, or *excellent (E)*. For analysis, these categorical ratings were converted to numerical values of 0%, 25%, 50%, 75%, and 100%, with all items weighted equally. Students were required to accurately satisfy at least half of the sub-items in each marking item to achieve a MS, and all the sub-items to achieve a CS. The quality of technique and efficiency was the key differentiator between a CS and E. A student’s performance was expressed as the average percentage score across all items.

#### Post-tutorial survey

Following the post-test, students were invited to complete a structured post-hoc questionnaire to capture their perceptions of the tutorial. The survey included five domains:

- Learning experience.
- Teaching effectiveness.
- Perceived utility of the session.
- Student engagement and participation.
- Anticipated long-term impact and knowledge retention.

Closed-ended items were measured on a five-point Likert scale (1 = strongly disagree to 5 = strongly agree). In addition, open-ended questions were included to allow participants to provide qualitative feedback on the strengths, weaknesses, and overall value of the tutorial.

### Data analysis

Data obtained from the pre-test, post-test and post-intervention questionnaire were subjected to statistical analysis. Descriptive results are reported using mean and standard deviation (SD). The paired samples *t-*test was used to determine statistical significance in the differences exhibited in student performance between the pre-test and post-test. All differences that were deemed to be statistically significant were defined with a *p-*value of less than 0.05. All the data was analysed using IBM SPSS Statistics (Version 30).

Responses to the open-ended survey items were analysed using a content analysis approach, following the method described by Hsieh and Shannon in 2005 [39]. Two researchers (KQ and BMA) independently reviewed and coded the written response, identified recurring ideas and themes, and resolved discrepancies through discussion.

### Reflexivity

The researchers brought complementary perspectives to the qualitative analysis. KQ, the student investigator, contributed an insider perspective from recent experience as a learner. BMA, an experienced medical educator, contributed expertise in teaching and assessment. This combination allowed for a more nuanced interpretation while acknowledging the potential for interpretive bias.

### Human ethics and consent to participate

Ethics approval for this study was gained through the University of Newcastle Human Research Ethics Committee (HREC-QA286). All participants in this study provided informed consent prior to enrolment. This study was conducted in accordance with the ethical standards as outlined in the Declaration of Helsinki.

## Results

Twenty-three second-year medical students enrolled in the UON JMP participated in this project during Semester 1 of 2025. All students completed the pre-test, tutorial and post-test. Seventeen (73.9%) out of 23 students completed the post-hoc questionnaire.

### Pre- and post-intervention assessment

The overall average performance of the participant cohort also observed a statistically significant improvement (pre-test: 63.8 ± 15.8 vs. post-test: 76.3 ± 10.5, *p* = 0.01).

Improvements were observed in 14 of the 15 assessment items when comparing the mean percentages of the post-test to the pre-test. Statistically significant improvements were seen in assessment items including “elbow palpation” (pre-test: 40.2% ± 28.9% vs. post-test: 62.0% ± 27.0%, *p* = 0.01), “shoulder palpation (pre-test: 41.3 ± 26.8 vs. post-test: 71.7 ± 23.0, *p* = 0.0002), “shoulder active range of motion” (pre-test: 63.0 ± 33.6 vs. post-test: 87.0 ± 14.8, *p* = 0.002), “shoulder passive range of motion” (pre-test: 57.6 ± 34.1 vs. post-test: 82.6 ± 20.6, *p =* 0.001), “performs examination without causing pain, distress or embarrassment” (pre-test: 94.6 ± 10.5 vs. post-test: 100.0 ± 0, *p =* 0.02), “organised and efficient examination in nominated timeframe” (pre-test: 54.3 ± 17.9 vs. post-test: 64.1 ± 19.7, *p* = 0.025) (Table 1).

**Table 1:**
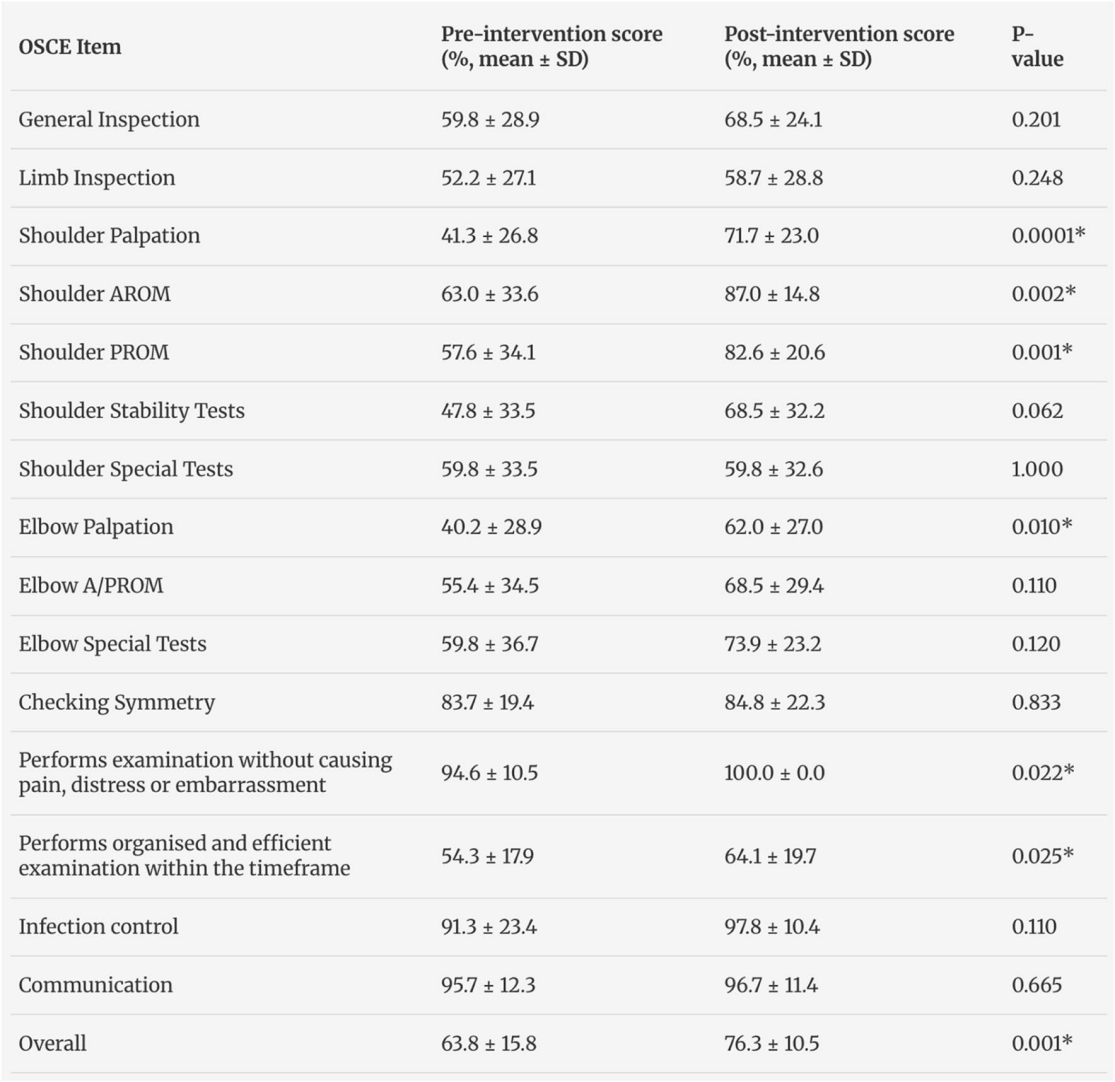
Student performance in the pre-test and post-test

*SD* Standard deviation, *AROM* Active range of motion, *PROM* Passive range of motion

^*^ Statistically significant

### Post-test questionnaire

Seventeen students (74% of participants) completed the post-test questionnaire. Their responses highlighted improvements in confidence, strong perceptions of teaching effectiveness, and an appreciation of the tutorial’s practical relevance.

### Learning experience

**Fig. 1:**
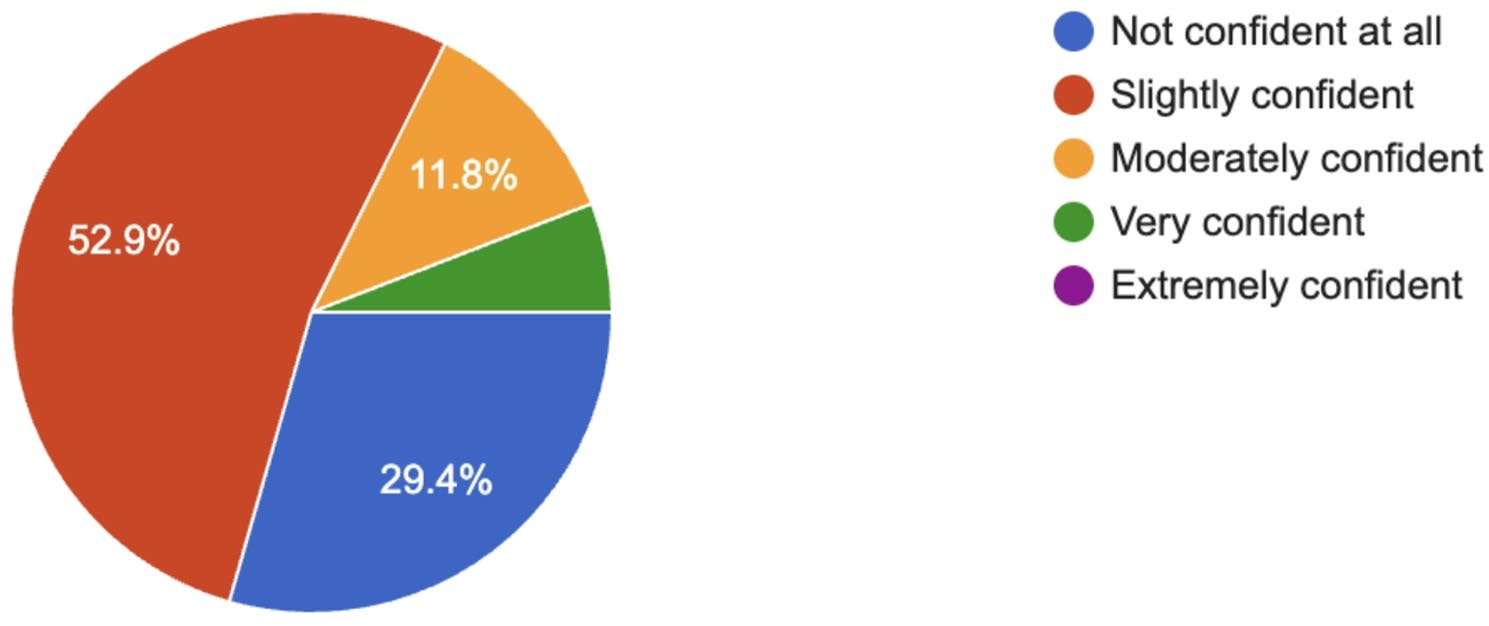
Responses to the question “How confident did you feel about performing an upper limb musculoskeletal examination before this session?”

**Fig. 2:**
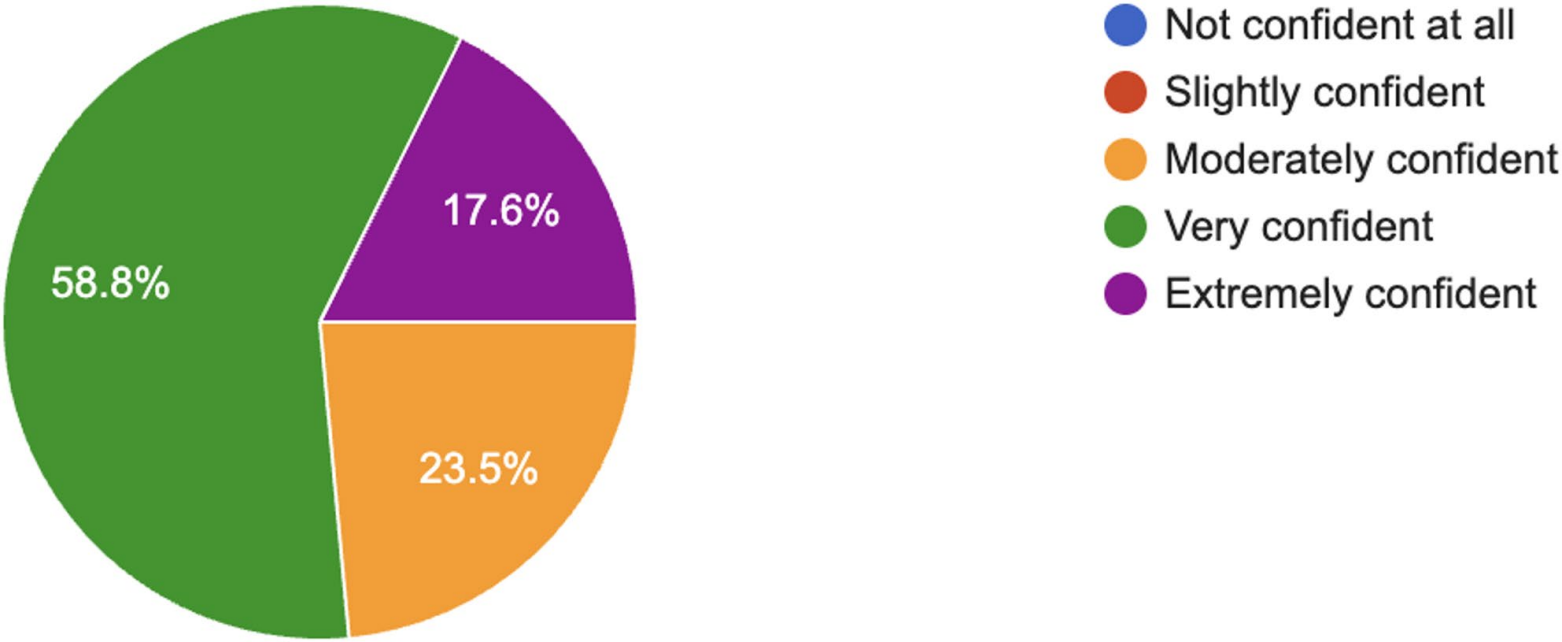
Responses to the question “How confident do you feel about performing an upper limb musculoskeletal examination after this session?”

**Fig. 3:**
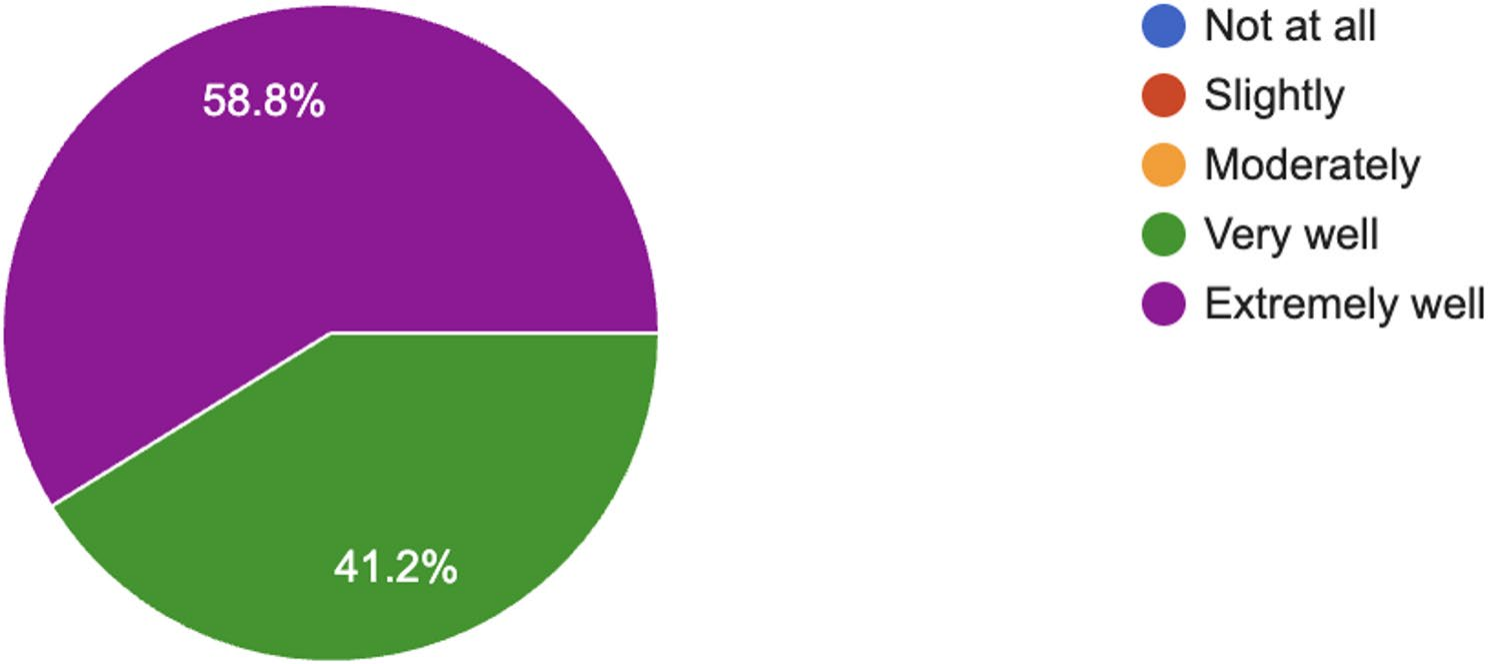
Responses to the question “How well did this session enhance your understanding of the upper limb musculoskeletal examination?”

Before the tutorial, most students reported limited confidence in performing an upper limb musculoskeletal examination: 9 (52.9%) felt *slightly confident* and 5 (29.4%) felt *not confident at all* (Fig. 1). Following the tutorial, levels of self-reported confidence improved markedly, with 10 (58.8%) students feeling *very confident*, 4 (23.5%) *moderately confident*, and 3 (17.6%) *extremely confident* (Fig. 2). Similarly, when asked “How well did this session enhance your understanding of the upper limb musculoskeletal examination?”, 10 (58.8%) reported *extremely well* and 7 (41.2%) reported *very well* (Fig. 3).

### Teaching effectiveness

**Fig. 4:**
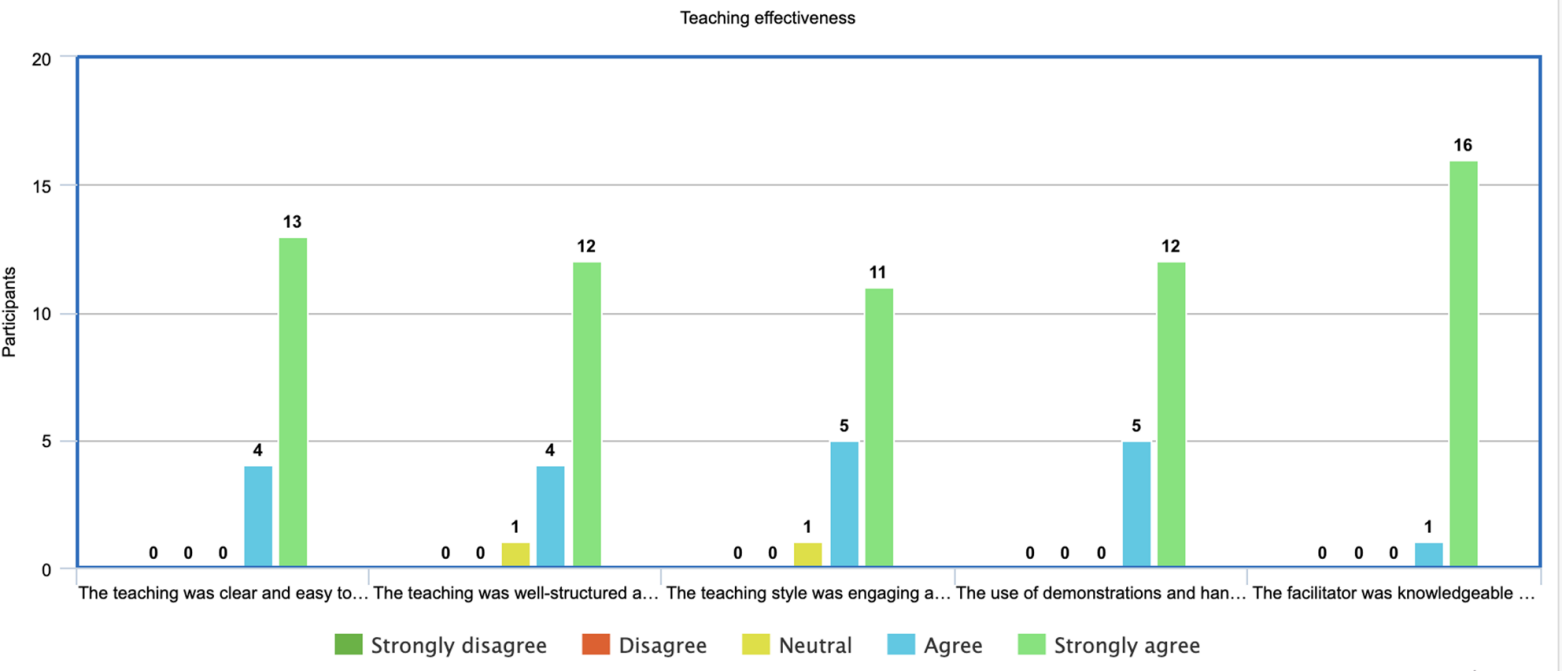
The statements on the x-axis of Fig. 4 from left to right are as follows. The teaching was clear and easy to understand. The teaching was well-structured and logically organised. The teaching was interactive and engaging. The use of demonstrations and hands-on practice were beneficial. The facilitator was knowledgeable and answered questions effectively

Students consistently rated the teaching approach highly. All respondents agreed that the session was clear and easy to understand, with 13 (76%) selecting *strongly agree* and 4 (24%) *agree*. Similarly, 12 (71%) *strongly agreed* that the tutorial was well-structured and logically organised, while 4 (24%) *agreed* and 1 (6%) remained neutral. Most found the teaching style engaging: 11 (65%) *strongly agreed*, 5 (29%) *agreed*, and 1 (6%) was neutral. The use of demonstration and hands-on practice was particularly valued, with 12 (71%) *strongly agreeing* and 5 (29%) *agreeing* that this enhanced their learning. Almost all respondents (94%) *strongly agreed* that the facilitator was knowledgeable and answered questions effectively (Fig. 4).

### Perceived utility

**Fig. 5:**
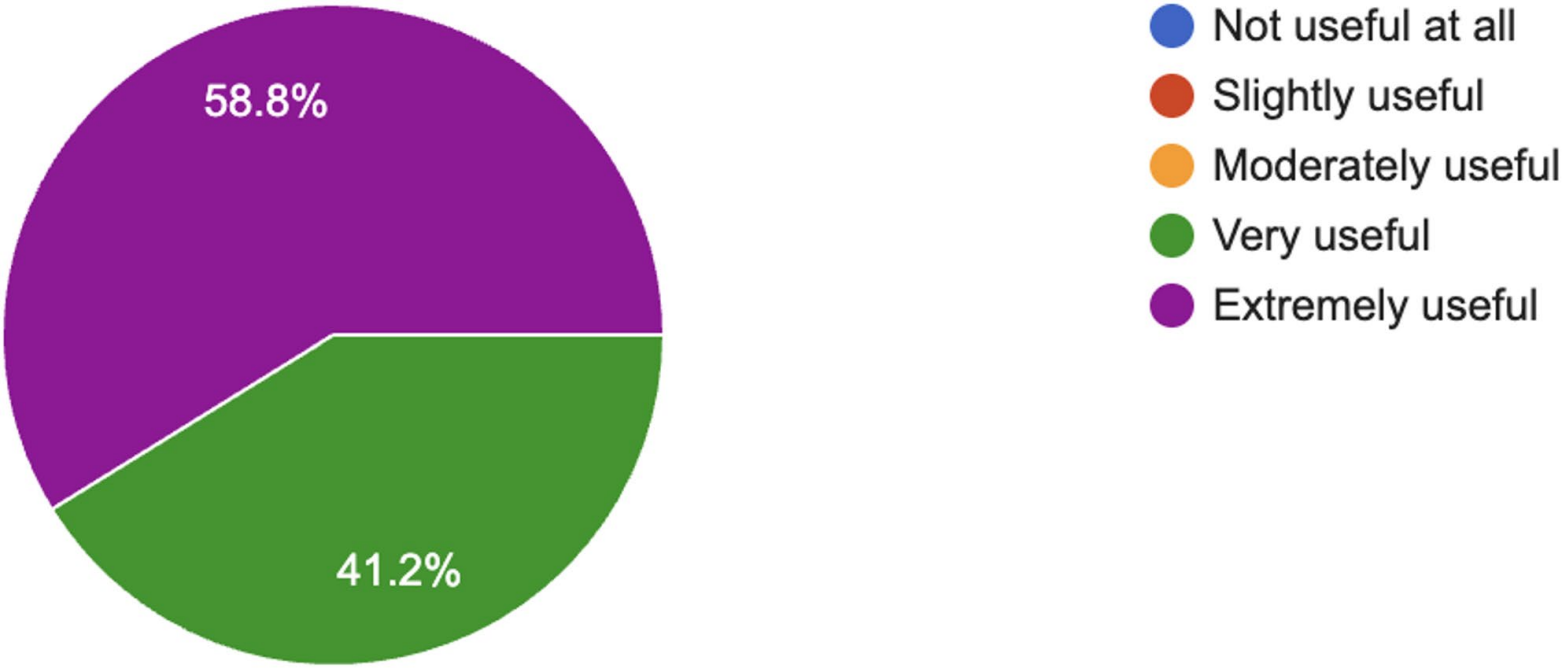
Responses to the question “How useful was this session in preparing you for future clinical practice?”

**Fig. 6:**
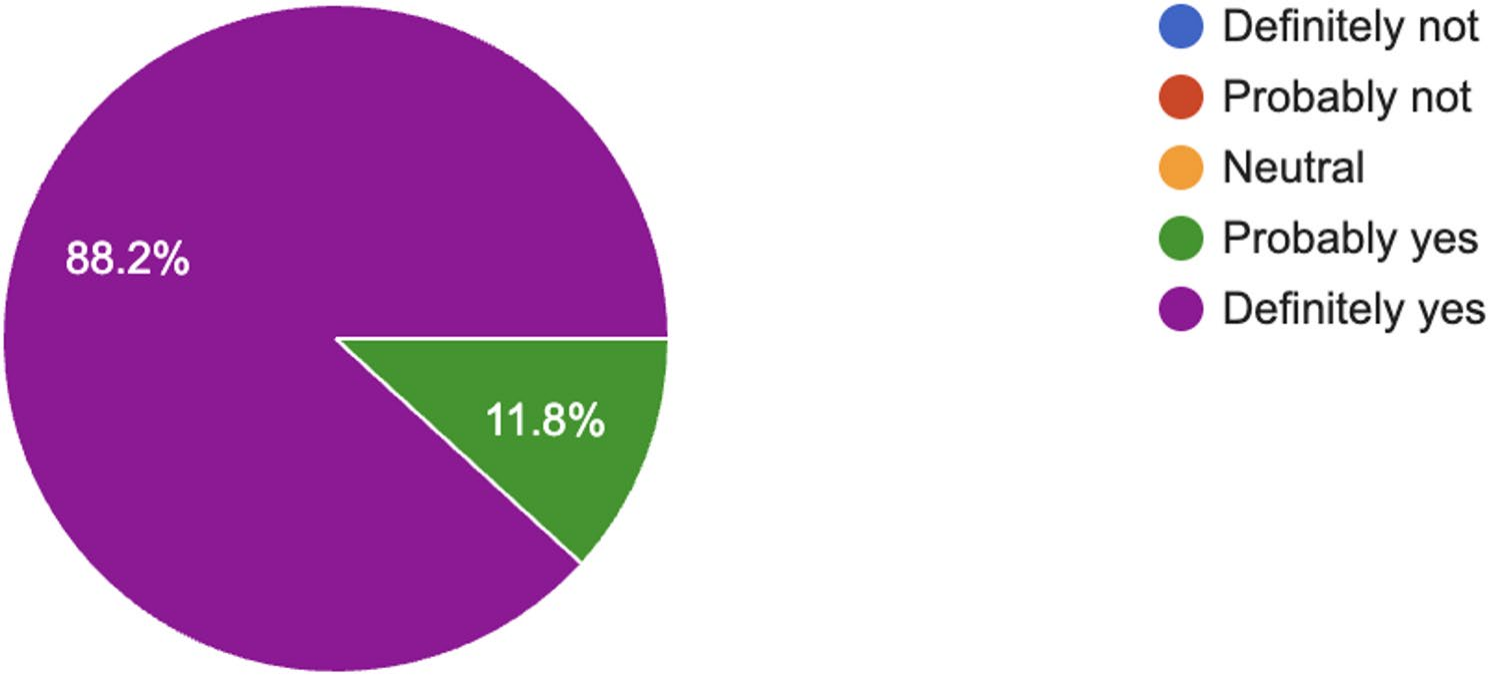
Responses to the question “Would you recommend this teaching session to other students?”

Students perceived the tutorial as directly relevant to their clinical training. Ten (59%) rated it as *extremely useful* in preparing them for future clinical practice, while 7 (41%) rated it *very useful* (Fig. 5). Almost all (15; 88%) reported they would “definitely” recommend the tutorial to peers, while the remainder (2; 12%) said they would “probably” recommend it (Fig. 6).

### Student engagement and participation

**Fig. 7:**
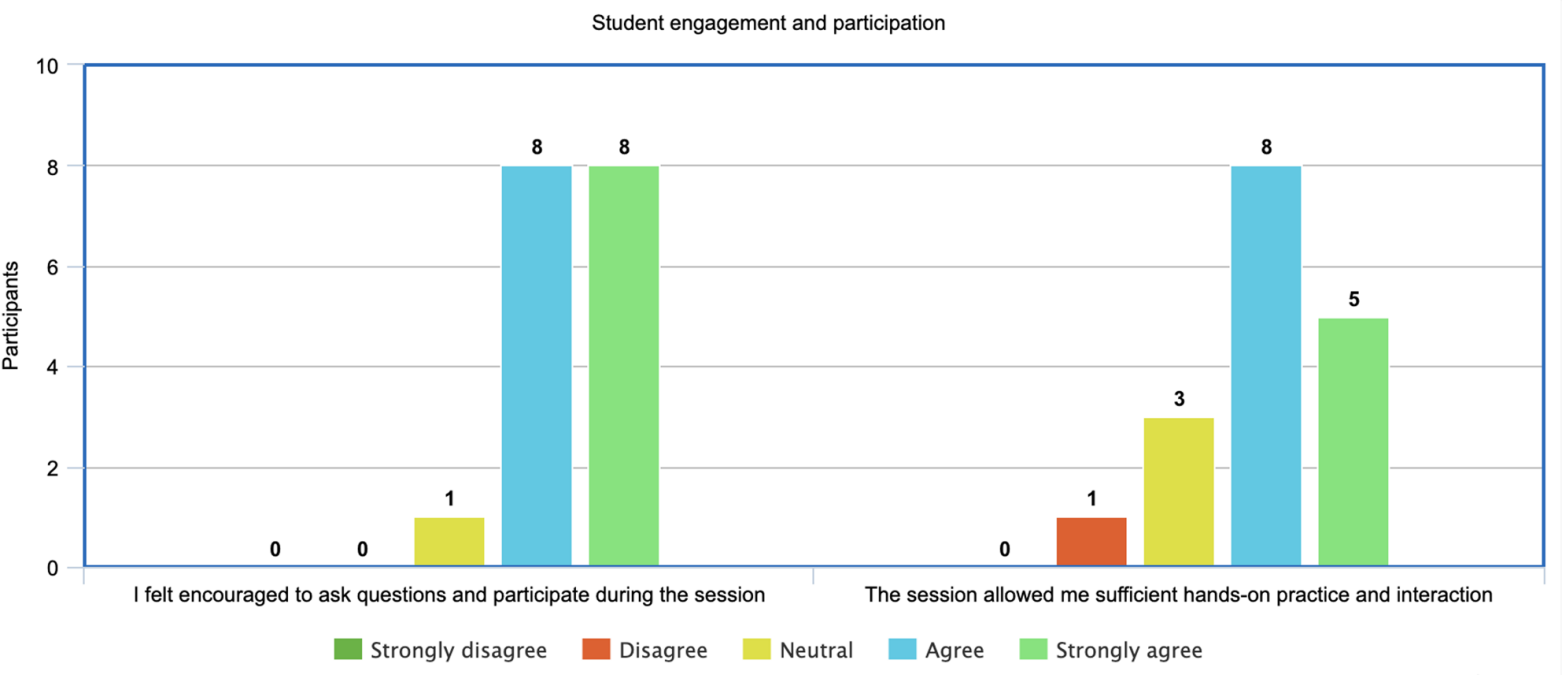
Student Engagement and Participation

Most students felt encouraged to participate, with 8 (47%) *strongly agreeing* and another 8 (47%) *agreeing* that they felt comfortable asking questions and contributing to the session. Only 1 student (6%) was neutral. Responses regarding hands-on practice were more mixed: 5 (29%) *strongly agreed* that sufficient practice time was provided, 8 (47%) *agreed*, 3 (18%) were neutral, and 1 (6%) disagreed (Fig. 7).

### Long term impact and retention

Eight students (47%) strongly agreed that they would be able to retain and apply the knowledge acquired from the teaching session in their clinical practice. Nine students (53%) agreed with this statement. Eleven students (65%) strongly agreed that they would benefit from a similar revision session in the future, while 5 students (29%) agreed and 1 (6%) remained neutral (Fig. 8).

**Figure:**
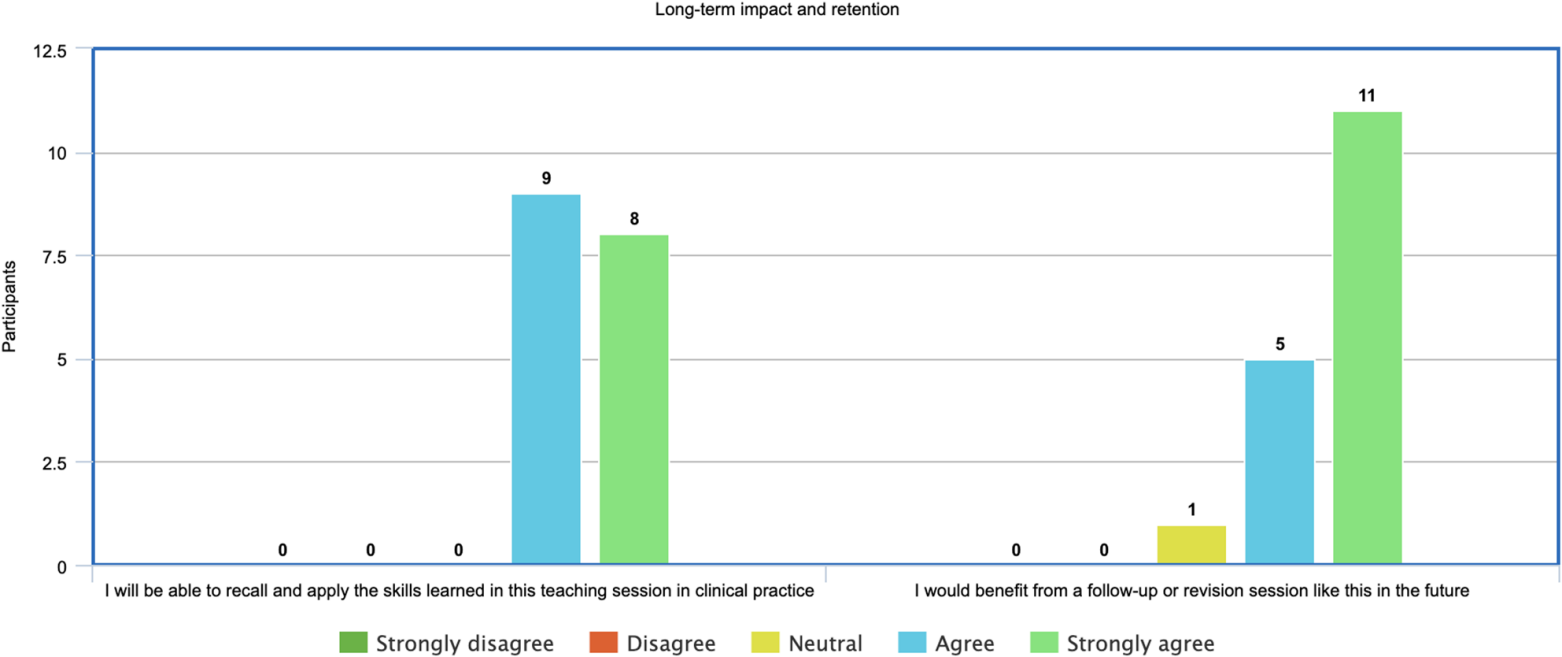
Fig. 8

### Open-ended survey responses

Analysis of open-ended survey responses revealed seven themes reflecting what students valued most about the near-peer-led tutorial and their suggestions for improvement.

#### Logical organisation

Students consistently emphasised the clarity and systematic nature of the session. Five of the 17 students (29%) highlighted the structured progression of the upper limb examination as a strength which provided a reproducible framework for practice. One student described the “organisation and detail of each section of the exam,” while another noted the “practical and logical ordering in which the examination was taught.” Others reinforced that the “logical structuring of the upper limb exam” enhanced their confidence in approaching the task systematically.

#### Conceptual clarity

When asked directly about the clarity of the tutorial, all but one student reported that the teaching was clear and well explained. The single exception suggested that clearer distinctions could be made between examination components that were formally assessed in the OSCE rubric and those that were clinically relevant but not explicitly marked. Despite this, students overwhelmingly endorsed the clarity of explanations and the logical step-by-step approach taken during the session.

#### Clinical reasoning and relevance

A central strength identified by students was the integration of clinical reasoning into the teaching. Learners reported that linking each manoeuvre to its diagnostic purpose deepened their understanding. One student reflected that they valued “learning about the clinical relevance behind each of the tests we are asked to perform,” while another emphasised the importance of “connecting how the movements are conducted and why, I often feel disconnected between these two things.” The explanation of pathologies associated with specific tests helped students understand not only how to perform them but also what they were testing for.

#### Relation to pathology and clinically relevant anatomy

Four out of the 17 (23.5%) students appreciated the incorporation of anatomy, particularly when connected to pathology. Students appreciated the “clear sectional breakdown of the anatomy which was easy to follow and understand” and the “level of detail in discussing surface anatomy.” These elements helped them contextualise examination findings within a clinically meaningful framework.

#### Demonstration

Demonstrations by the near-peer tutor were described as a pivotal part of the learning experience. Observing the examination performed correctly after attempting it themselves allowed students to identify and correct errors. As one participant explained: “Seeing the upper limb examination performed correctly soon after we performed it made me more aware of the common errors and the correct technique.” Others valued learning special test techniques “which weren’t specifically covered in the clinical skills session.” This combination of demonstration and practice reinforced correct technique and provided exposure to extension topics.

#### Knowledge consolidation

Sixteen of the 17 (94%) students reported that the tutorial consolidated prior knowledge and reduced the perceived complexity of the examination. The stepwise approach was praised for reinforcing understanding: “Going through each part step-by-step and checking our understanding along the way helped reinforce the content and made it feel less overwhelming.” This method allowed students to integrate new learning with existing knowledge in a structured and manageable way.

#### Dedicated time for hands-on practice

Although students valued the explanations and demonstrations, many emphasised the importance of hands-on practice. One participant highlighted the benefit of “allocated time during the session to practise the correct techniques,” while another commented on “having a section of the session where we can try each of the movements on peers.” Some also expressed a desire for additional practice opportunities: “Would have been useful to practice and consolidate the learning and resolve any confusion.” These comments reinforce the role of repetition and rehearsal in building confidence and competence.

#### Overall perception

All students reported that the near-peer tutorial was superior to traditional learning resources such as lectures, textbooks, or videos. They explained that the interactive and practice-based format provided a depth of understanding and engagement that static or passive learning methods could not replicate. Furthermore, all students reported that the tutorial improved their ability to perform a thorough and effective upper limb musculoskeletal examination in the clinical setting, underscoring its practical educational value.

## Discussion

Recognising the underrepresentation of musculoskeletal teaching in medical curricula [6–9], this study evaluated a near-peer-led OSCE-style tutorial to determine its effectiveness in improving students’ examination performance and confidence, while also capturing their reflections on its educational value. The study demonstrated significant gains in students’ upper limb musculoskeletal examination skills after a near-peer tutorial. However, these improvements should be interpreted within the context of a short follow-up interval which reflects immediate recall and confidence. Such improvements are important given the high burden of musculoskeletal disease in Australia – for example, about 7.3 million Australians (≈ 29% of the population) live with chronic musculoskeletal conditions [40]. By bridging gaps in standard curricula, near-peer teaching (NPT) offers the potential to improve clinical competency. Consistent with peer-assisted learning theory, these outcomes may be explained by cognitive and social congruence [21]. In cognitive congruence, tutors share a similar knowledge base with learners, allowing them to explain examination techniques in familiar language [41]. Social congruence means the tutor and student occupy similar social roles (e.g. fellow medical students), creating a friendly, non-judgmental learning environment [20, 42]. In such an environment, students feel less anxious and more willing to ask questions and practice skills. Our participants’ large post-test gains in palpation and range-of-motion tasks suggest that peer tutors were able to present these concepts at an accessible level, likely reducing students’ cognitive load and encouraging active participation [43]. By contrast, complex special tests showed little change, likely because mastery of nuanced manoeuvres requires repeated clinical practice beyond a single session. This aligns with prior studies, which found that technically demanding skills such as special tests often require repeated, spaced exposure to be mastered [44, 45].

The intervention also exemplified experiential learning. According to Kolb’s model, effective learning involves a cycle of concrete experience, reflection, abstract conceptualisation, and active experimentation. The near-peer tutorial gave students a concrete experience – hands-on practice of examination manoeuvres – immediately followed by feedback and discussion. This integration of practice with explanation aligns with experiential learning principles, which have been shown to strengthen clinical skills acquisition [46]. Successfully performing exam tasks likely provided students with mastery experiences, which, according to Bandura’s self-efficacy theory, enhance learners’ confidence in their abilities. Indeed, mastery and the resulting self-efficacy gains may have driven improved performance on the post-test [47, 48]. In our study, students’ nearly perfect scores on maintaining patient comfort and communication suggest that the experience bolstered both their competence and confidence in conducting respectful exams. In summary, the NPT approach delivered learning that was active, reflective, and confidence-building – hallmarks of experiential learning that likely translated into higher post-test scores [30].

The tutor involved in this intervention also reported improved self-confidence, communication skills, and motivation to continue teaching. This aligns with prior work showing that acting as a peer tutor enhances clinical mastery, self-efficacy, and educator identity [47]. We believe this bidirectional benefit warrants further investigation.

These findings echo prior research on near-peer and peer-assisted learning. For example, Schiff and colleagues reported that medical students who participated in a resident-led, near-peer musculoskeletal curriculum scored roughly 30% higher on a standardised musculoskeletal knowledge test after the intervention [48]. Similarly, in an anatomy OSCE context, Rashid et al. found that 73.2% of students considered teaching by recent graduates to be comparable to consultant-led teaching [28]. In our study, the overwhelmingly positive student feedback mirrored these results: learners valued the tutorial’s relevance and clarity. Collectively, the literature suggests that well-prepared near-peer instructors – whether senior students or junior doctors – can improve tutee knowledge and skills in a way that is acceptable and appropriate to learners [28, 48].

### Practical implications

To our knowledge, this study represents the first evaluation of the effectiveness and learner perspectives on medical student–facilitated near-peer teaching (NPT) in musculoskeletal education within an Australian medical school. While our findings contribute to the growing body of literature on peer-assisted learning, we acknowledge that similar interventions have been explored in other educational contexts.

Our results support the integration of NPT as a practical adjunct to faculty-led musculoskeletal education, particularly in pre-clinical years where students are developing foundational clinical skills. NPT may help address persistent gaps in undergraduate MSK training by offering additional opportunities for structured, hands-on practice in a socially and cognitively congruent environment.

Despite increasing interest in peer-led instruction, there remains a lack of systematic reviews or meta-analyses evaluating the role of NPT in musculoskeletal and orthopaedic education globally. A 2022 systematic review of musculoskeletal education in undergraduate medical curricula analysed 44 studies, only one of which compared changes in knowledge and clinical skills after teaching by musculoskeletal specialists versus non-specialists. Notably, none of the included studies investigated NPT as an intervention [49, 50]. This highlights the need for further empirical exploration of NPT in this field.

NPT also offers a feasible solution to the increasing time and workload constraints faced by senior clinical educators. Junior doctors and senior medical students can deliver high-quality, peer-relevant teaching without the scheduling burden typically associated with consultant-led sessions [51]. For instance, junior educators have successfully facilitated musculoskeletal teaching programs with high levels of student satisfaction. From the learner’s perspective, near-peer sessions may enhance engagement and reduce barriers to participation through shared social identity, relatability, and the creation of a low-stakes learning environment [14, 15].

Importantly, the benefits of NPT extend to the tutors themselves. Acting as peer educators has been shown to foster self-confidence and self-efficacy in clinical and teaching competencies. In our study, the student facilitator reported improved presentation skills and a strengthened motivation to teach, echoing findings from Avonts et al., who described increased self-efficacy among peer tutors [47]. Other studies have similarly demonstrated that NPT enhances the clinical knowledge, communication skills, and leadership capabilities of facilitators. For example, Medveczky et al. found that junior doctors involved in the Near Peer Medical Teaching (NPMT) initiative gained greater confidence in teaching and were more likely to pursue educational roles in the future [52].

Beyond knowledge consolidation, NPT promotes professional identity formation and develops key capabilities in communication, feedback, and leadership skills essential for clinicians who will take on teaching responsibilities throughout their careers [53–55]. Taken together, these findings suggest that NPT can serve as a “win-win” model in medical education, enhancing learner outcomes while also cultivating the next generation of clinical educators.

###  Strengths and limitations

A key strength of this study was its use of trained peer assessors (senior clinical-phase students who were also physiotherapy practitioners), which likely ensured consistent, expert feedback without overburdening faculty. The mixed-methods design (quantitative exam results plus qualitative feedback) provided insight into learning gains and a comprehensive view of student perceptions. Our cohort size was comparable to similar musculoskeletal NPT studies, allowing meaningful statistical comparison [48].

Several limitations should be noted. Firstly, a singular author facilitated the NPT session (KQ), participation was voluntary and the cohort was small which significantly limits the generalisability of the findings of this study. The absence of a control group restricts the ability to attribute improvements solely to the intervention and further limits the ability to deduce causal or comparative interpretations of the results. The pre-test, tutorial, and post-test all occurred within the same week; hence the improvements reflect short-term recall rather than sustained learning. Absence of randomisation or blinding introduces potential biases: assessors knew the students and anticipated improvement, and students may have prepared specifically for the observed post-test (a Hawthorne effect). Additionally, this study examined only a single near-peer session on one topic (upper limb exam). It remains unclear whether similar gains would be seen across multiple sessions or other musculoskeletal topics. Finally, baseline proficiency was already very high in areas like hygiene and communication, limiting observable gains in these assessment domains. Further research is warranted. Longitudinal studies should assess whether peer-led skills are retained over time and transferred to real clinical encounters. Expanding the curriculum to cover more joint exams and including larger, multi-centre cohorts would test generalisability. Future research may consider comparing NPT against other teaching modalities including formal faculty-led teaching. Ultimately, meta-analyses of musculoskeletal education programs are needed; to date most reviews of MSK teaching have not examined near-peer interventions [48]. If larger studies confirm our results, medical schools could confidently integrate NPT as a standard adjunct to bolster essential musculoskeletal competencies.

## Conclusion

This study demonstrates that a single, near-peer facilitated tutorial supports improvement in pre-clinical medical students’ objective performance and self-reported confidence in conducting the upper limb musculoskeletal examination. Framed by theories of cognitive and social congruence, experiential learning, and self-efficacy, the findings highlight near-peer teaching as a feasible adjunct to standard musculoskeletal curricula, with benefits for both learners and facilitators. Broader and longitudinal studies are warranted to confirm its long-term impact and generalisability.

## Supplementary Information

Supplementary Material 1.

Supplementary Material 2.

Supplementary Material 3.

## Data Availability

The data analysed and interpreted in this study is available upon reasonable request.
