# Supplementary material for: Enhancing musculoskeletal examination skills through near-peer teaching: student outcomes and perspectives
Source: BMC Med Educ. 2026 Apr 18;26:1012. doi: 10.1186/s12909-026-09235-2 (PMC13285311; doi:10.1186/s12909-026-09235-2)
Supplement: Supplementary file 1 — Supplementary Material 1. [file 12909_2026_9235_MOESM1_ESM.pdf]

Upper Limb Musculoskeletal Examination Teaching Session Feedback Questionnaire

Thank you for participating in my Health Pathways Education Research Project and upper limb musculoskeletal examination teaching session. Your feedback is valuable in evaluating the standard of my teaching and its utility to yourselves as students. Please take a few minutes to answer the following questions regarding the ***teaching session*** you received on the upper limb musculoskeletal examination.

* Indicates required question

Learning Experience

1. How confident did you feel about performing an upper limb musculoskeletal examination ***before*** this session? *

Mark only one oval.

- Not confident at all
- Slightly confident
- Moderately confident
- Very confident
- Extremely confident

2. How confident do you feel about performing an upper limb musculoskeletal examination ***after*** this session? *

Mark only one oval.

- Not confident at all
- Slightly confident
- Moderately confident
- Very confident
- Extremely confident

3. How well did this session enhance your understanding of the upper limb musculoskeletal examination? *

Mark only one oval.

- [ ] Not at all
- [ ] Slightly
- [ ] Moderately
- [ ] Very well
- [ ] Extremely well

Teaching Effectiveness

4. Please provide a rating for the following statements. *

Mark only one oval per row.

	Strongly disagree	Disagree	Neutral	Agree	Strongly agree
**The teaching was clear and easy to understand.**	◯	◯	◯	◯	◯
**The teaching session was well-structured and logically organised.**	◯	◯	◯	◯	◯
**The teaching style was engaging and interactive.**	◯	◯	◯	◯	◯
**The use of practical demonstrations and hands-on practice was beneficial.**	◯	◯	◯	◯	◯
**The facilitator was knowledgeable and answered questions effectively.**	◯	◯	◯	◯	◯

Perceived Utility

5. How useful was this session in preparing you for future clinical practice? *

Mark only one oval.

- Not useful at all
- Slightly useful
- Moderately useful
- Very useful
- Extremely useful

6. Would you recommend this teaching session to other students? *

Mark only one oval.

- Definitely not
- Probably not
- Neutral
- Probably yes
- Definitely yes

Student Engagement and Participation

7. Please provide a rating for the following statements. *

Mark only one oval per row.

	Strongly disagree	Disagree	Neutral	Agree	Strongly agree
**I felt encouraged to ask questions and participate during the session.**	◯	◯	◯	◯	◯
**The session allowed me sufficient hands-on practice and interaction.**	◯	◯	◯	◯	◯

Long Term Impact and Retention

8. Please provide a rating for the following statements. *

Mark only one oval per row.

	Strong disagree	Disagree	Neutral	Agree	Strongly agree
**I feel that I will be able to recall and apply the skills learned in this teaching session in future clinical practice.**	◯	◯	◯	◯	◯
**I would benefit from a follow-up or revision session like this in the future.**	◯	◯	◯	◯	◯

Open-ended questions

For the following questions, please be honest and as specific/detailed as you can with your justifications for your answers! Thank you.

9. What was the most helpful aspect of the session, and why? *

10. What part of the session do you think could be improved, and how? *

11. Was there anything unclear or difficult to understand during the session? If so, what would have helped clarify it? *

12. How did this session compare to the traditional methods of learning (e.g., lectures, textbooks, videos)? *

13. Do you feel that this session has improved your ability to perform an upper limb musculoskeletal examination in a real clinical setting? Why or why not? *

14. Would you be interested in a follow-up session or additional hands-on practice? If so, what topics would you like covered? *

15. Any additional comments, suggestions, or feedback?

This content is neither created nor endorsed by Google.

Google Forms
