# Supplementary material for: Enhancing musculoskeletal examination skills through near-peer teaching: student outcomes and perspectives
Source: BMC Med Educ. 2026 Apr 18;26:1012. doi: 10.1186/s12909-026-09235-2 (PMC13285311; doi:10.1186/s12909-026-09235-2)
Supplement: Supplementary file 2 — Supplementary Material 2. [file 12909_2026_9235_MOESM2_ESM.pptx]

## Slide 1
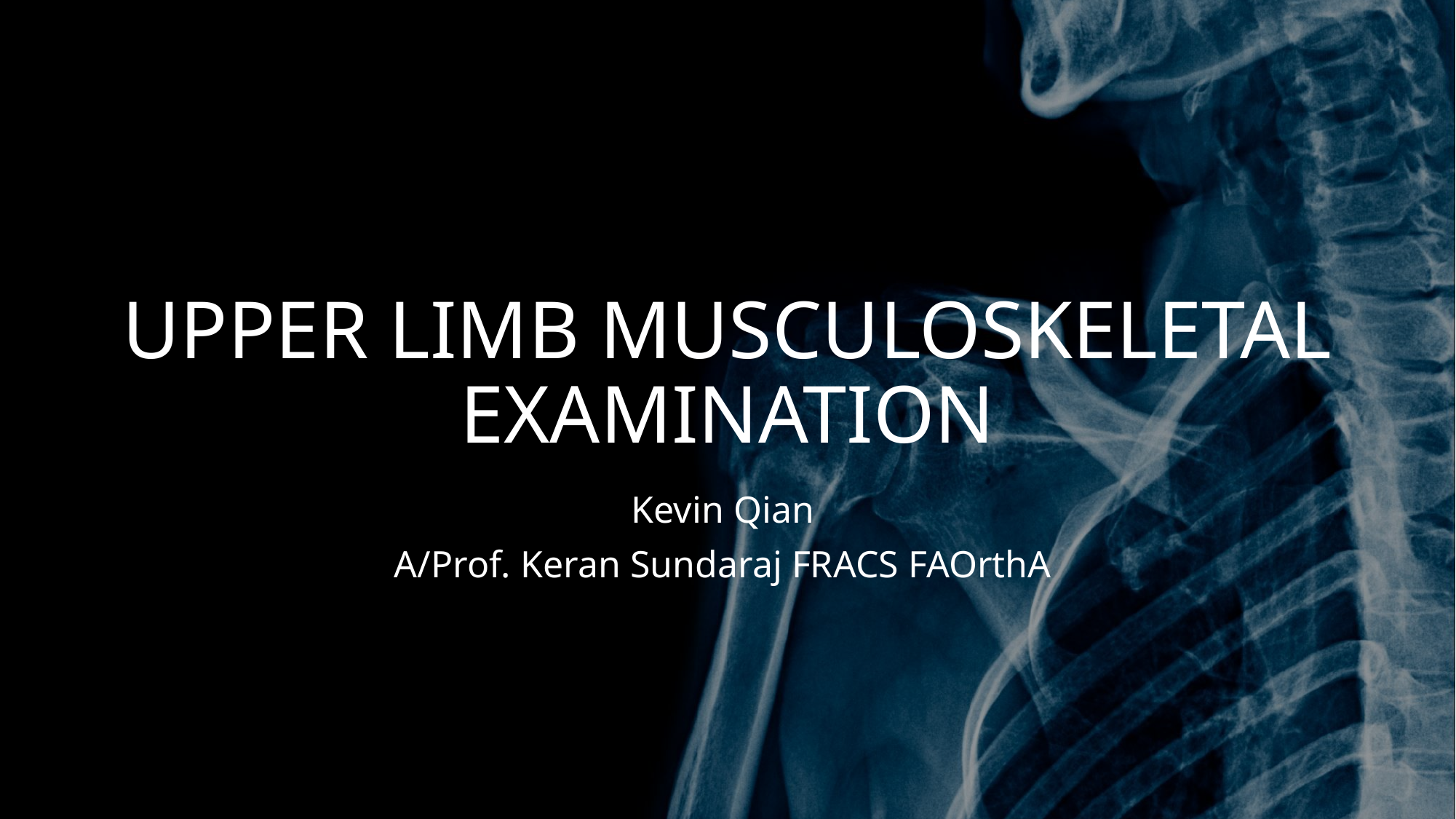

# UPPER LIMB MUSCULOSKELETAL EXAMINATION
Kevin Qian
A/Prof. Keran Sundaraj FRACS FAOrthA

## Slide 2
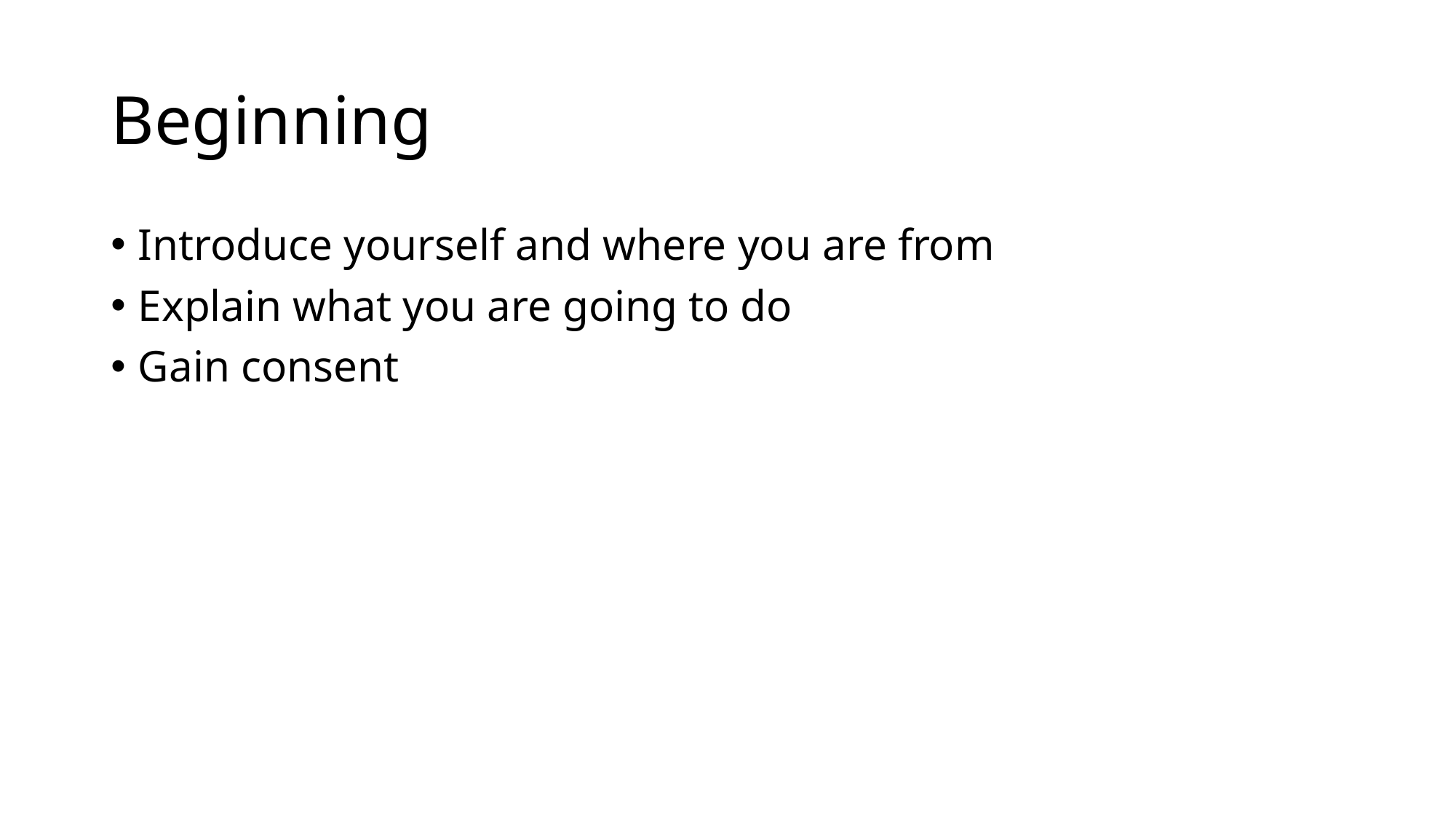

# Beginning
Introduce yourself and where you are from
Explain what you are going to do
Gain consent

## Slide 3
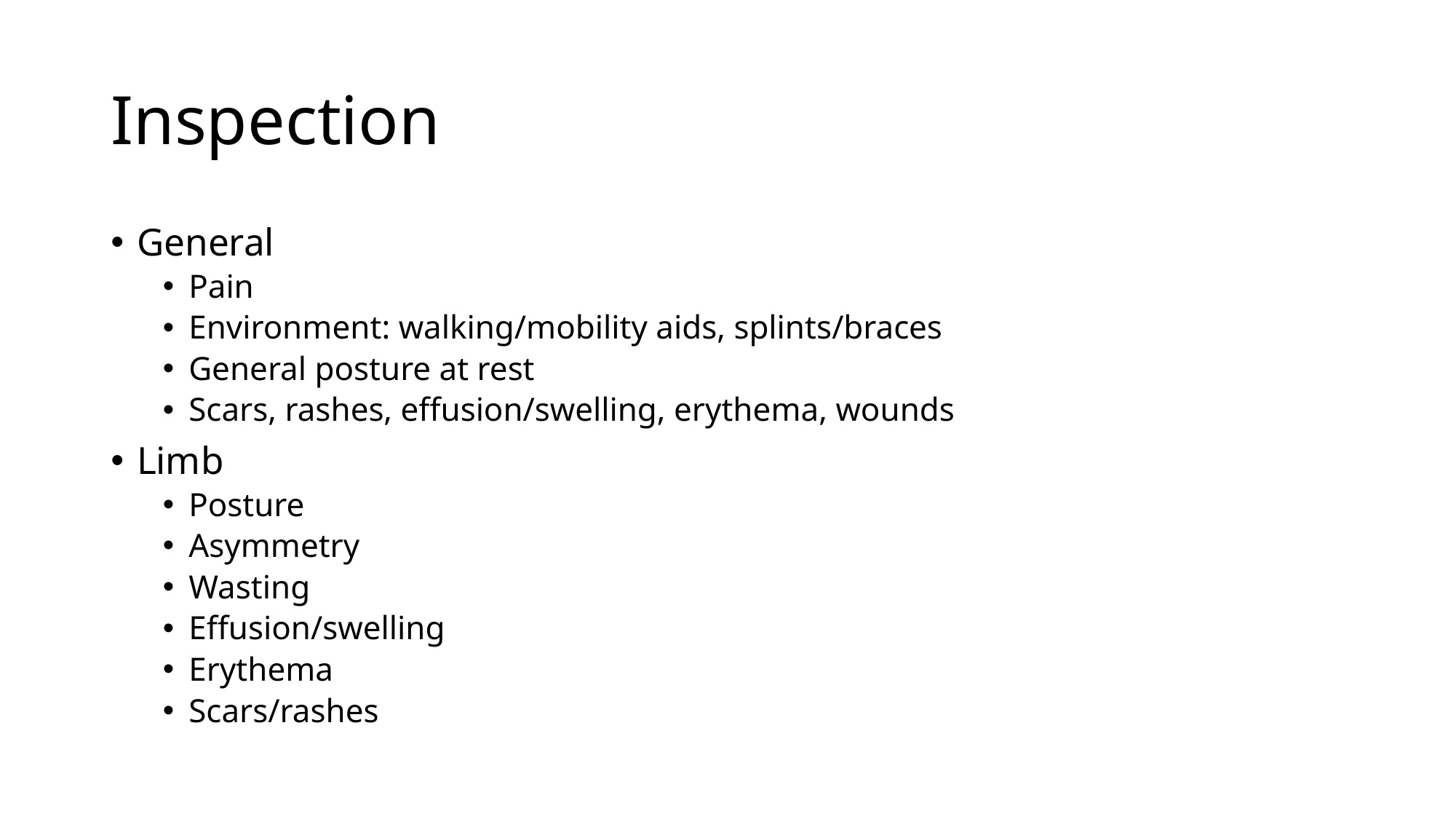

# Inspection
General
Pain
Environment: walking/mobility aids, splints/braces
General posture at rest
Scars, rashes, effusion/swelling, erythema, wounds
Limb
Posture
Asymmetry
Wasting
Effusion/swelling
Erythema
Scars/rashes

## Slide 4
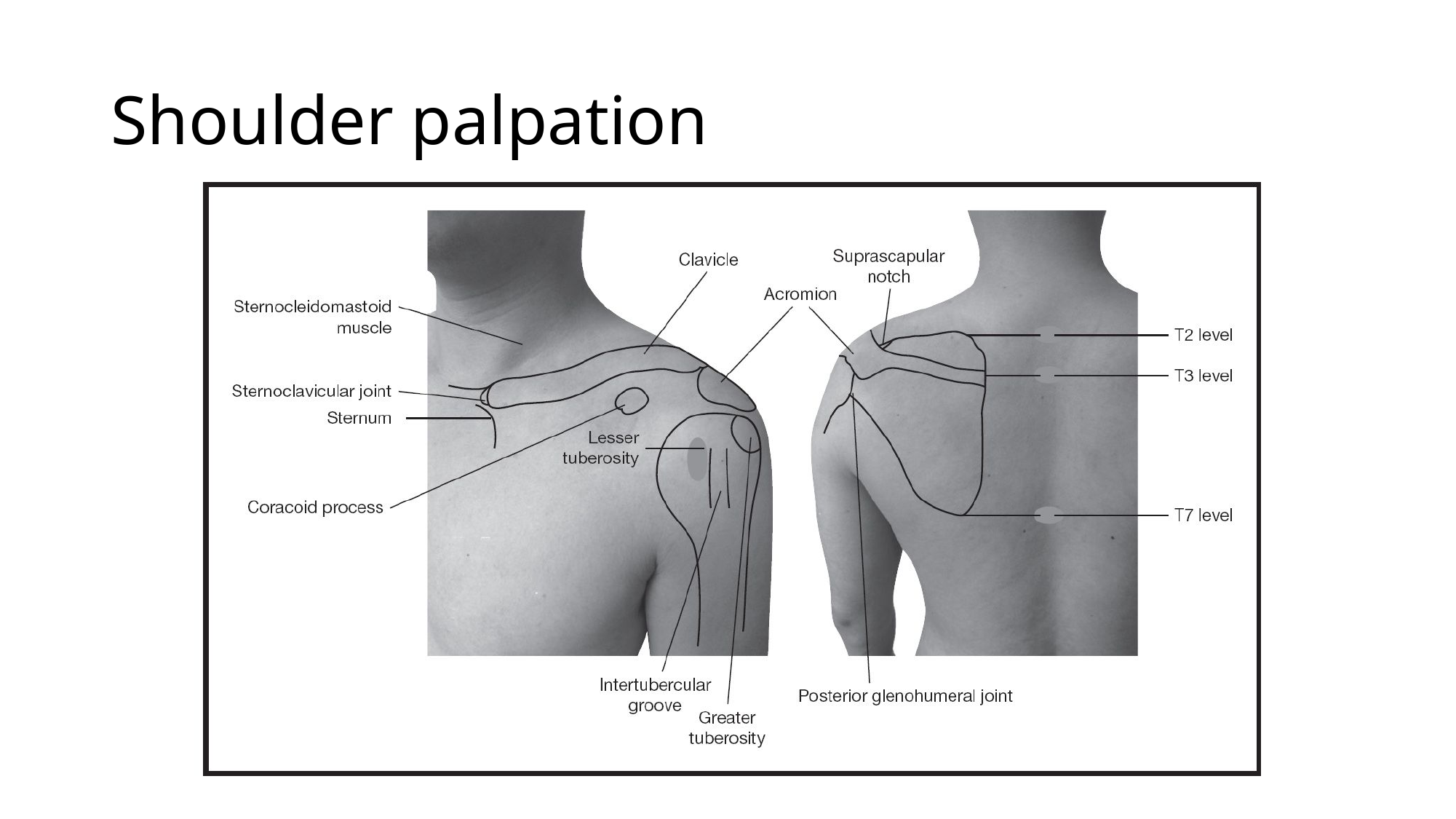

# Shoulder palpation

## Slide 5
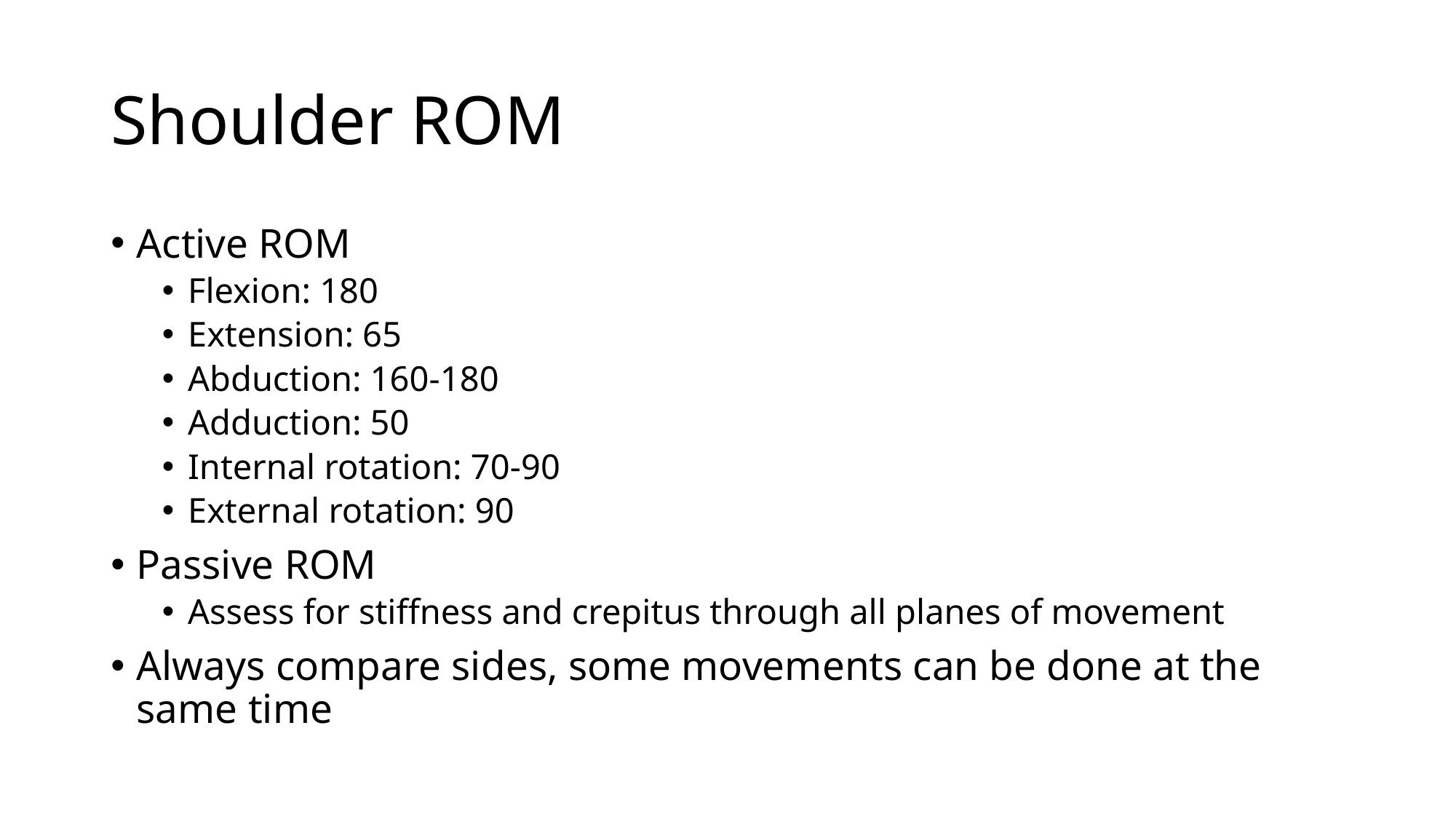

# Shoulder ROM
Active ROM
Flexion: 180
Extension: 65
Abduction: 160-180
Adduction: 50
Internal rotation: 70-90
External rotation: 90
Passive ROM
Assess for stiffness and crepitus through all planes of movement
Always compare sides, some movements can be done at the same time

## Slide 6
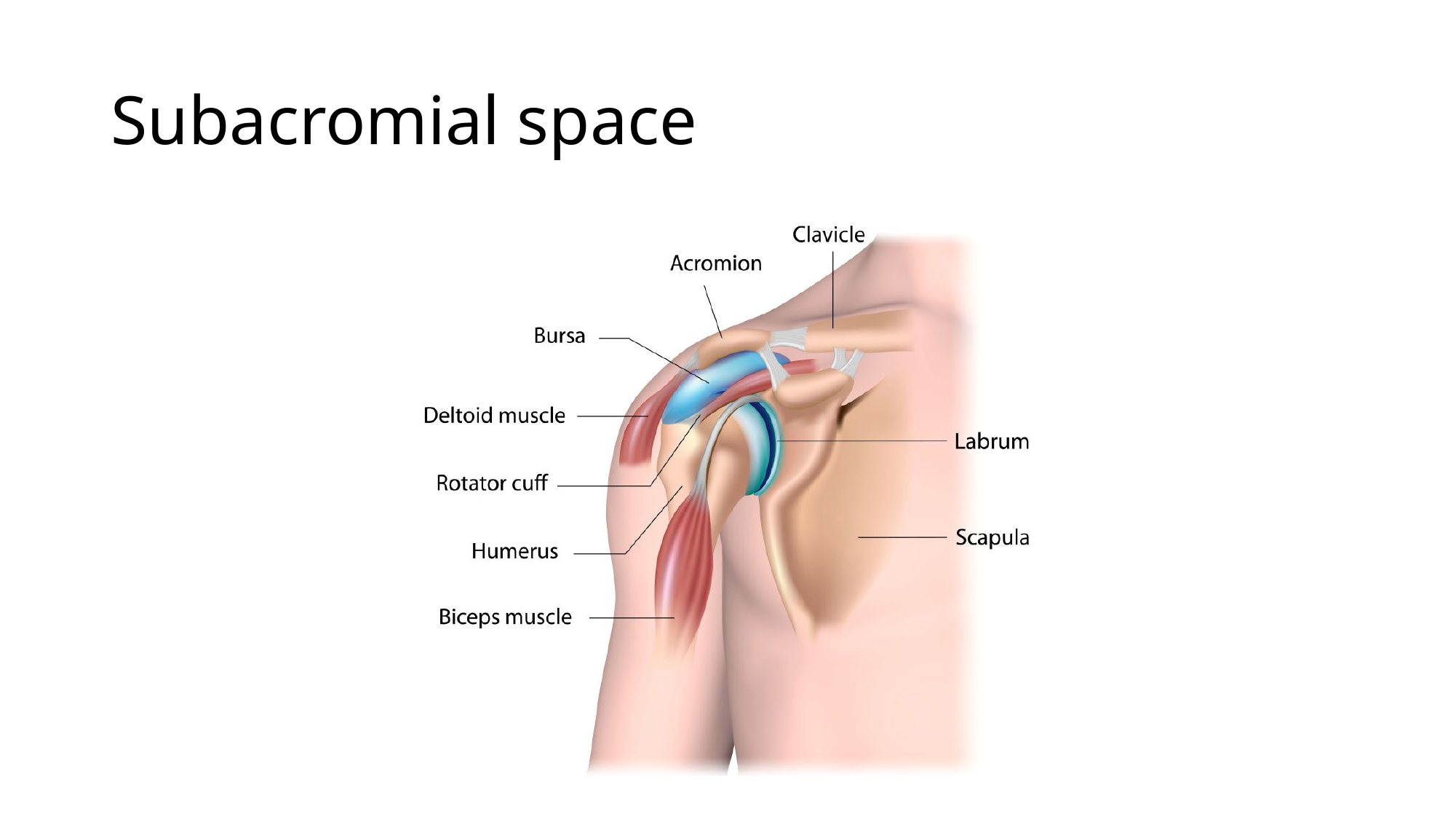

# Subacromial space

## Slide 7
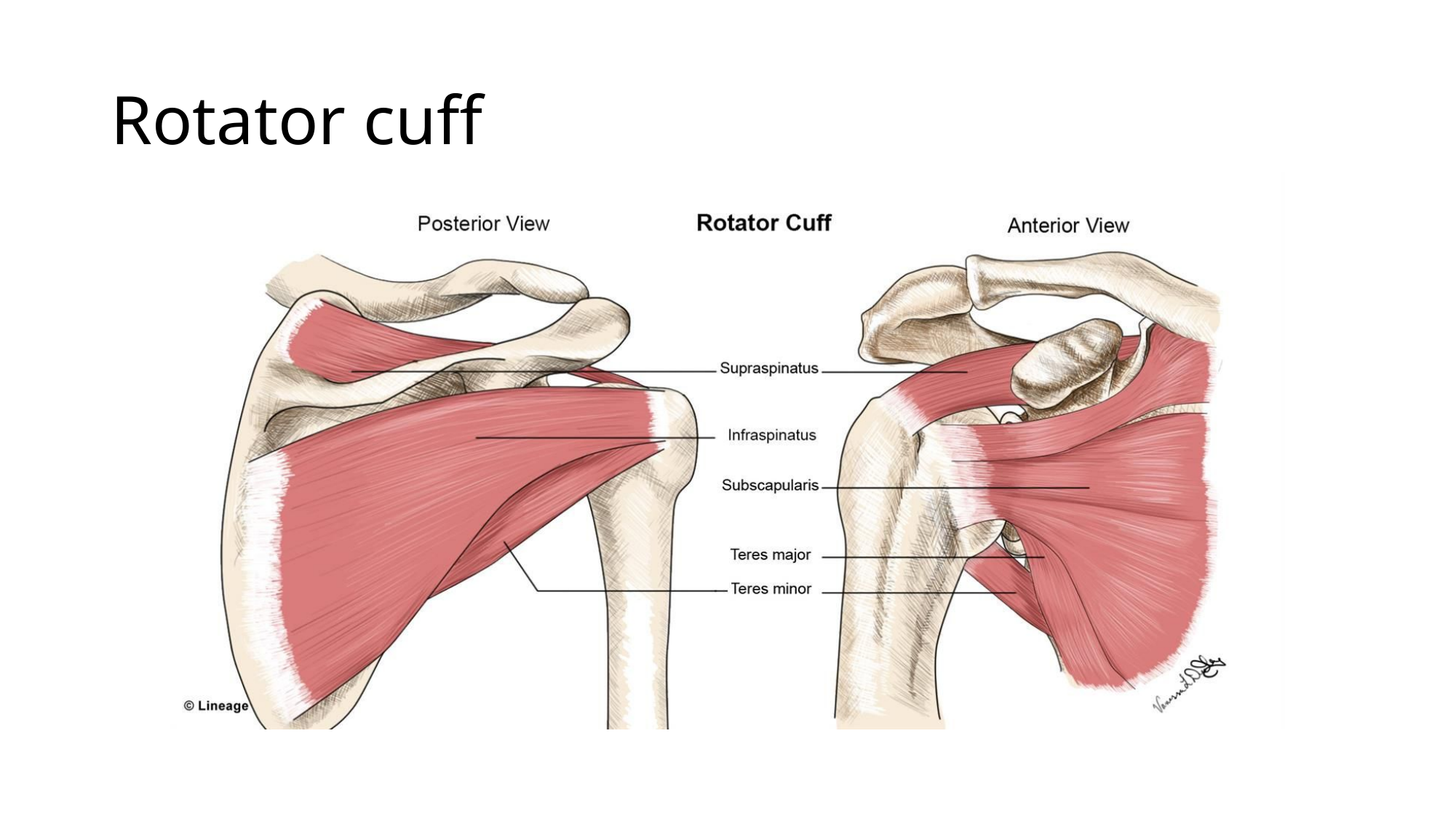

# Rotator cuff

## Slide 8
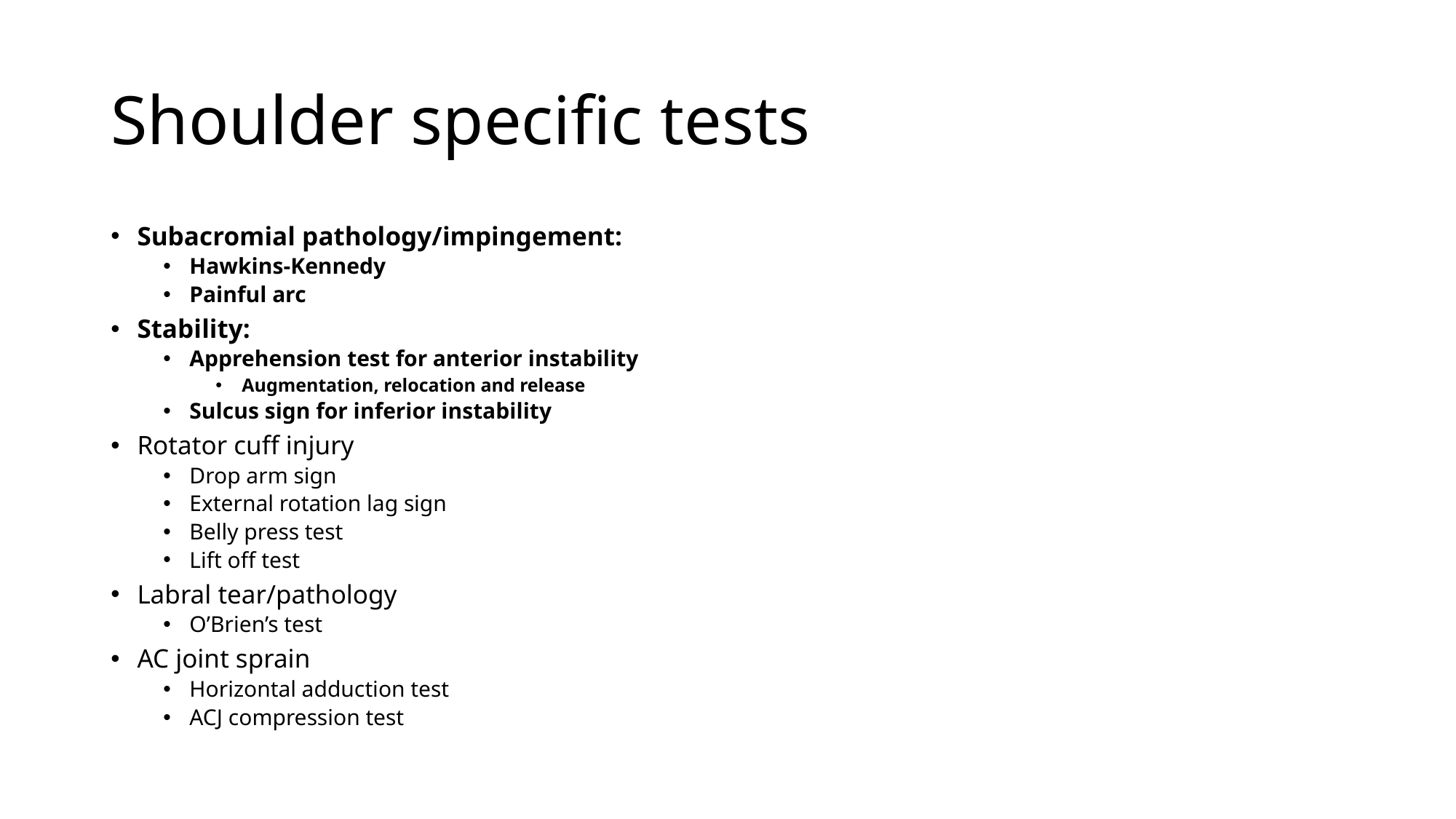

# Shoulder specific tests
Subacromial pathology/impingement:
Hawkins-Kennedy
Painful arc
Stability:
Apprehension test for anterior instability
Augmentation, relocation and release
Sulcus sign for inferior instability
Rotator cuff injury
Drop arm sign
External rotation lag sign
Belly press test
Lift off test
Labral tear/pathology
O’Brien’s test
AC joint sprain
Horizontal adduction test
ACJ compression test

## Slide 9
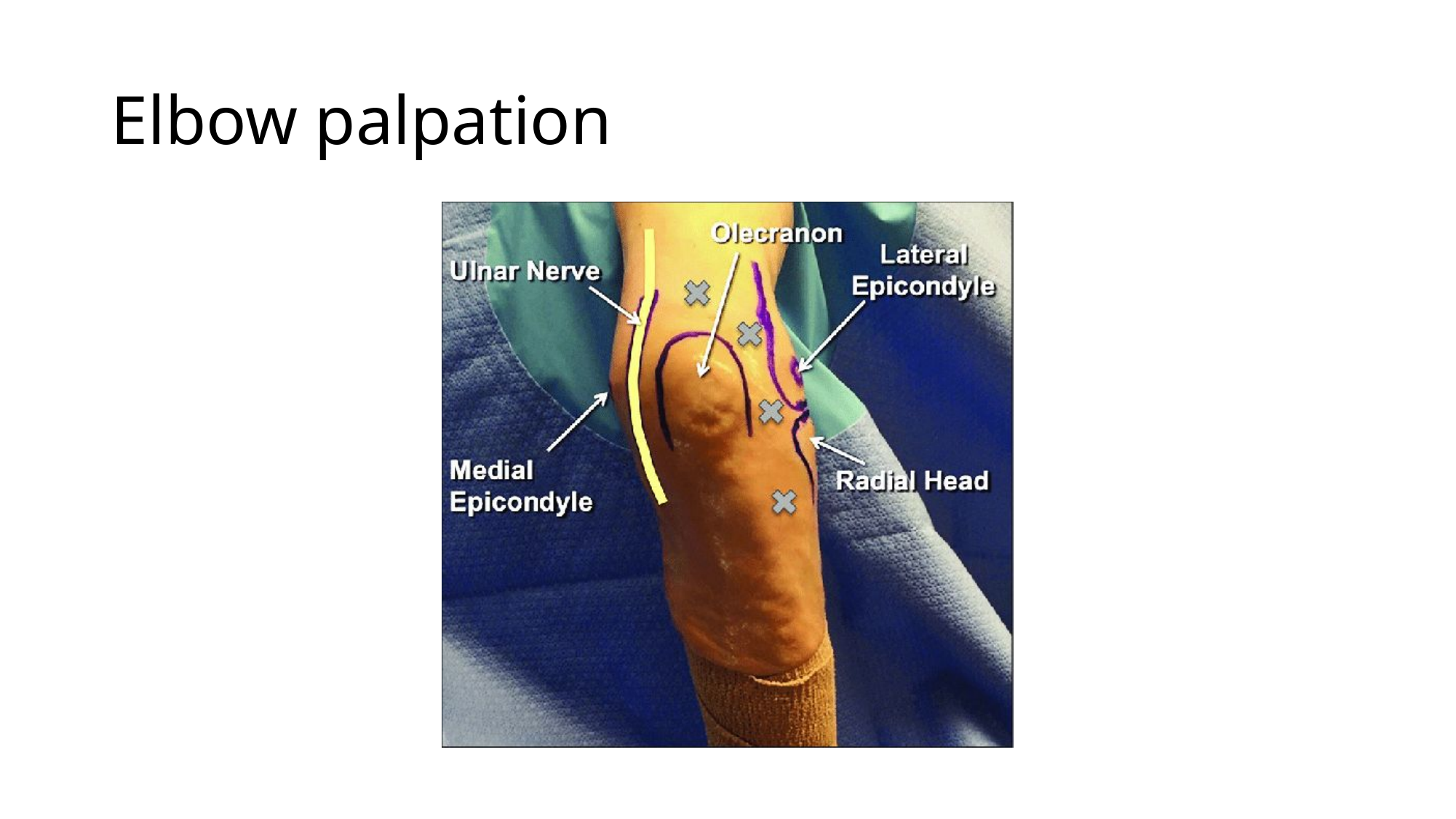

# Elbow palpation

## Slide 10
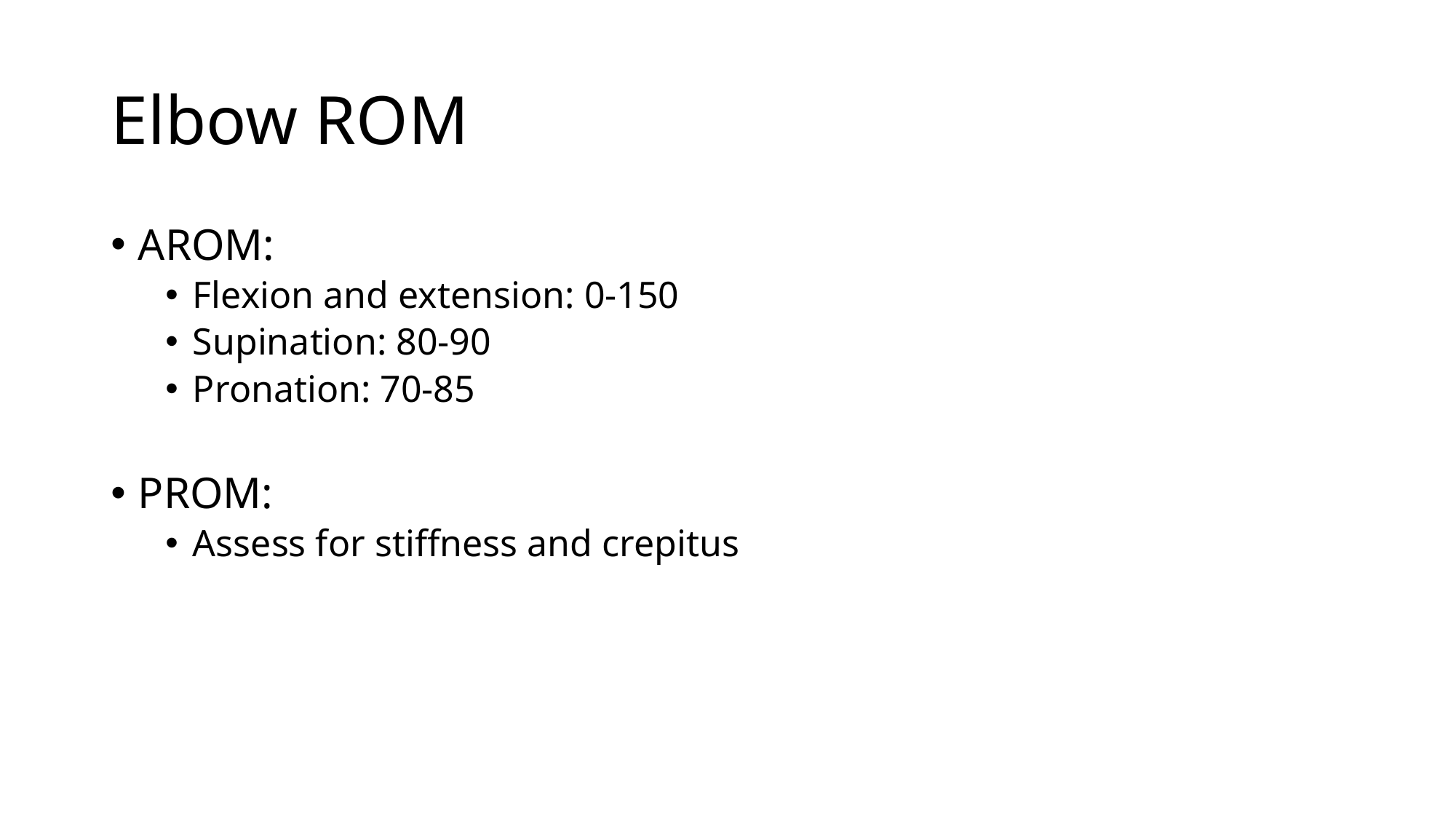

# Elbow ROM
AROM:
Flexion and extension: 0-150
Supination: 80-90
Pronation: 70-85
PROM:
Assess for stiffness and crepitus

## Slide 11
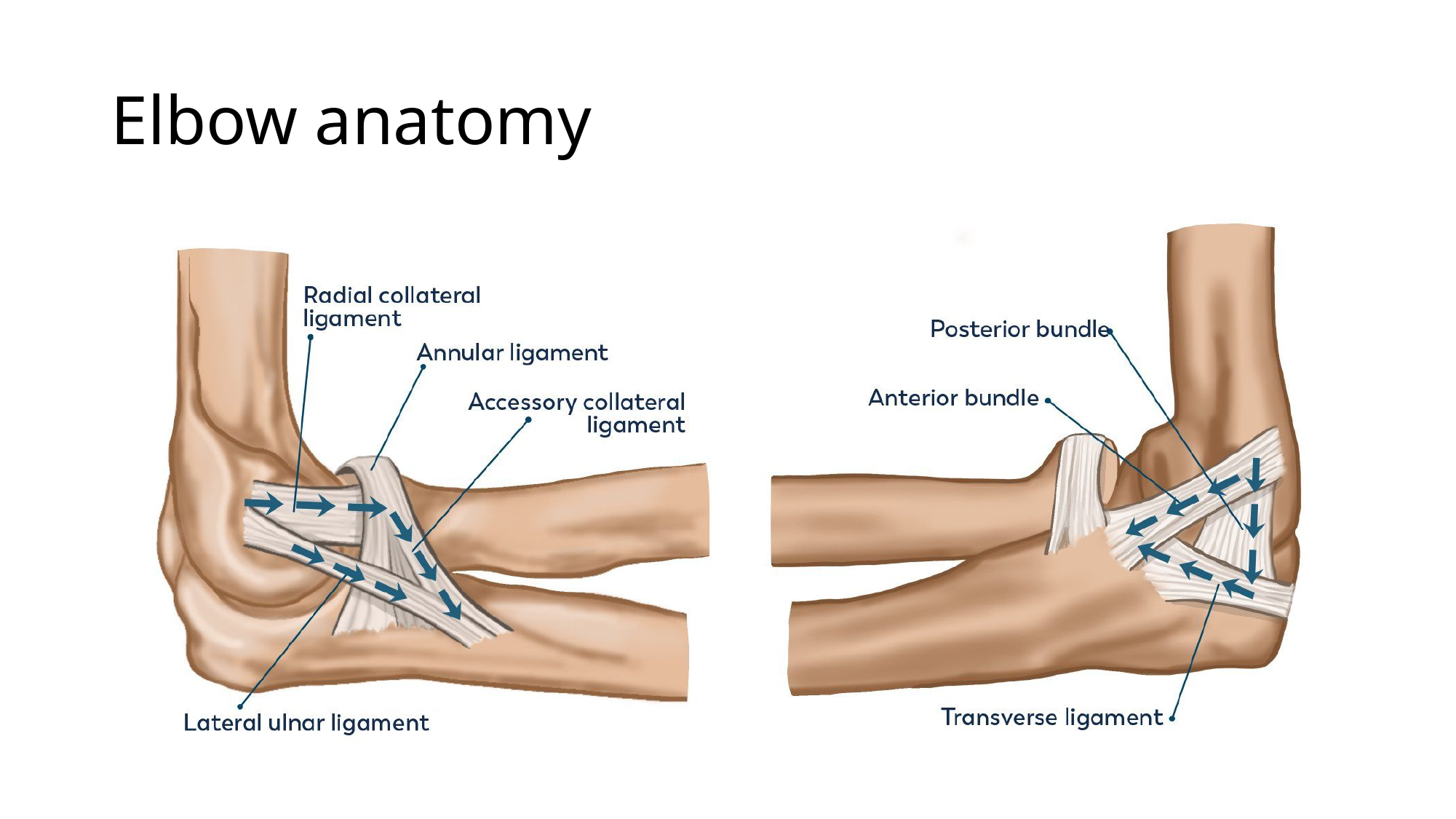

# Elbow anatomy

## Slide 12
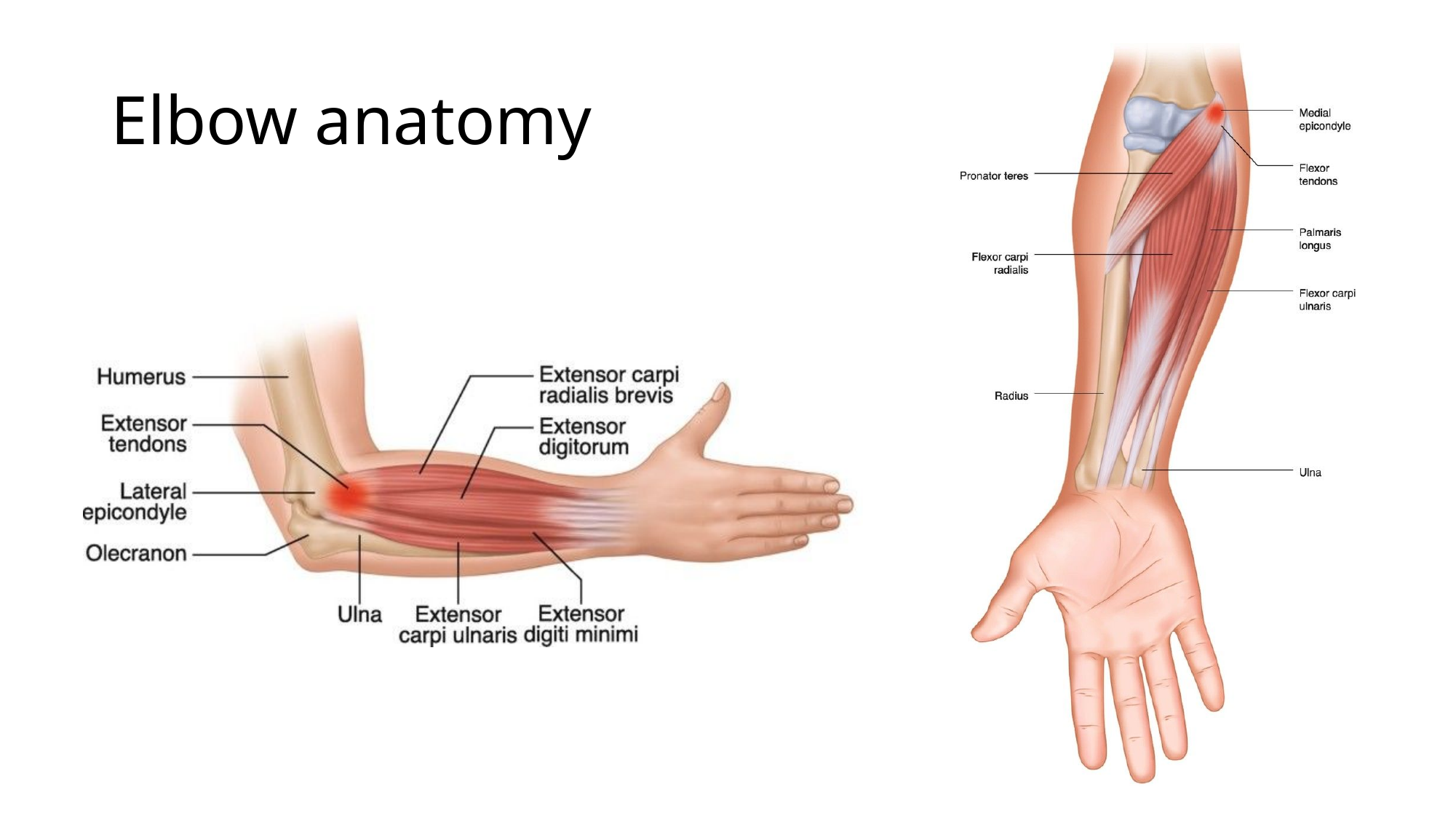

# Elbow anatomy

## Slide 13
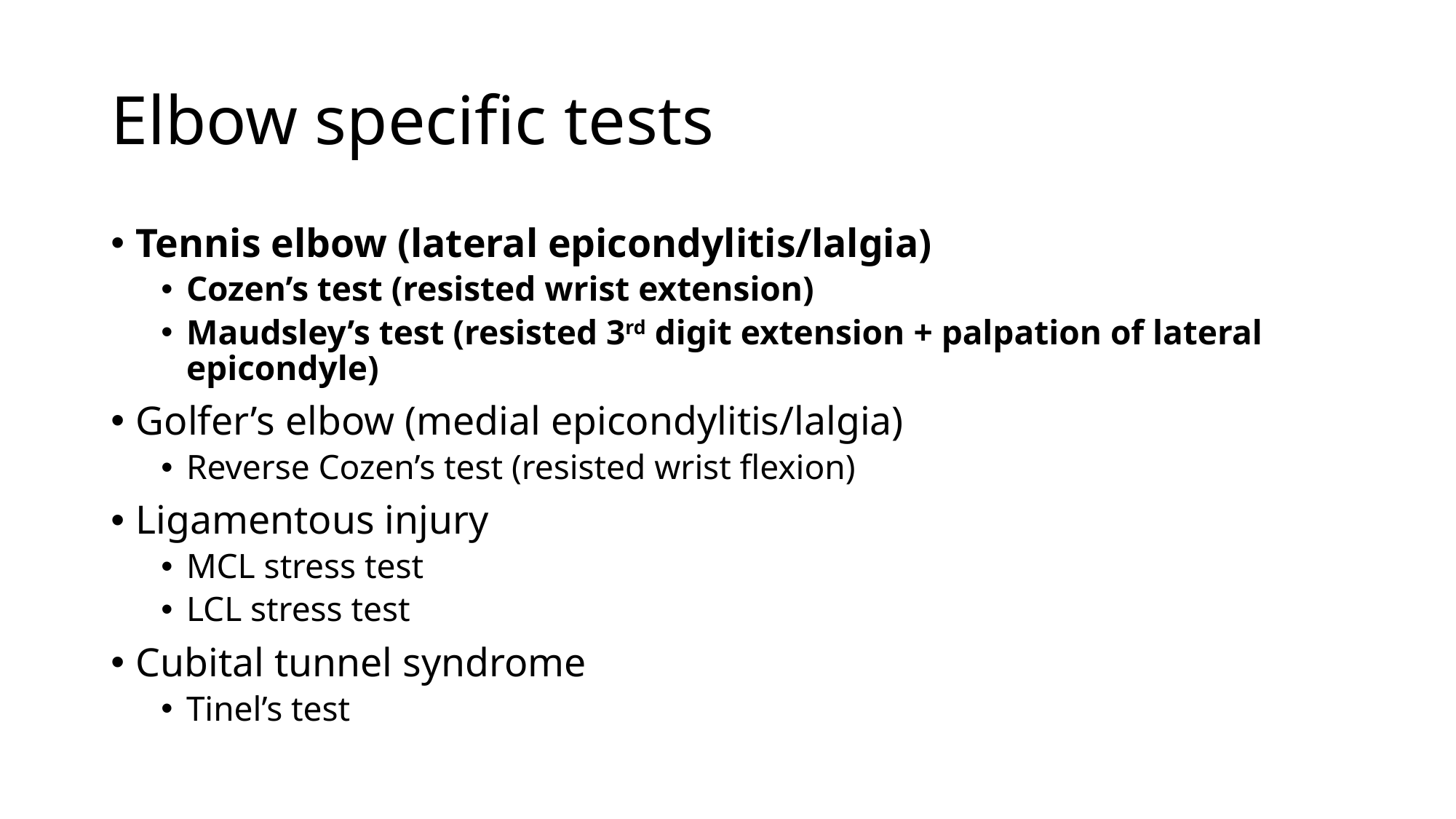

# Elbow specific tests
Tennis elbow (lateral epicondylitis/lalgia)
Cozen’s test (resisted wrist extension)
Maudsley’s test (resisted 3rd digit extension + palpation of lateral epicondyle)
Golfer’s elbow (medial epicondylitis/lalgia)
Reverse Cozen’s test (resisted wrist flexion)
Ligamentous injury
MCL stress test
LCL stress test
Cubital tunnel syndrome
Tinel’s test

## Slide 14
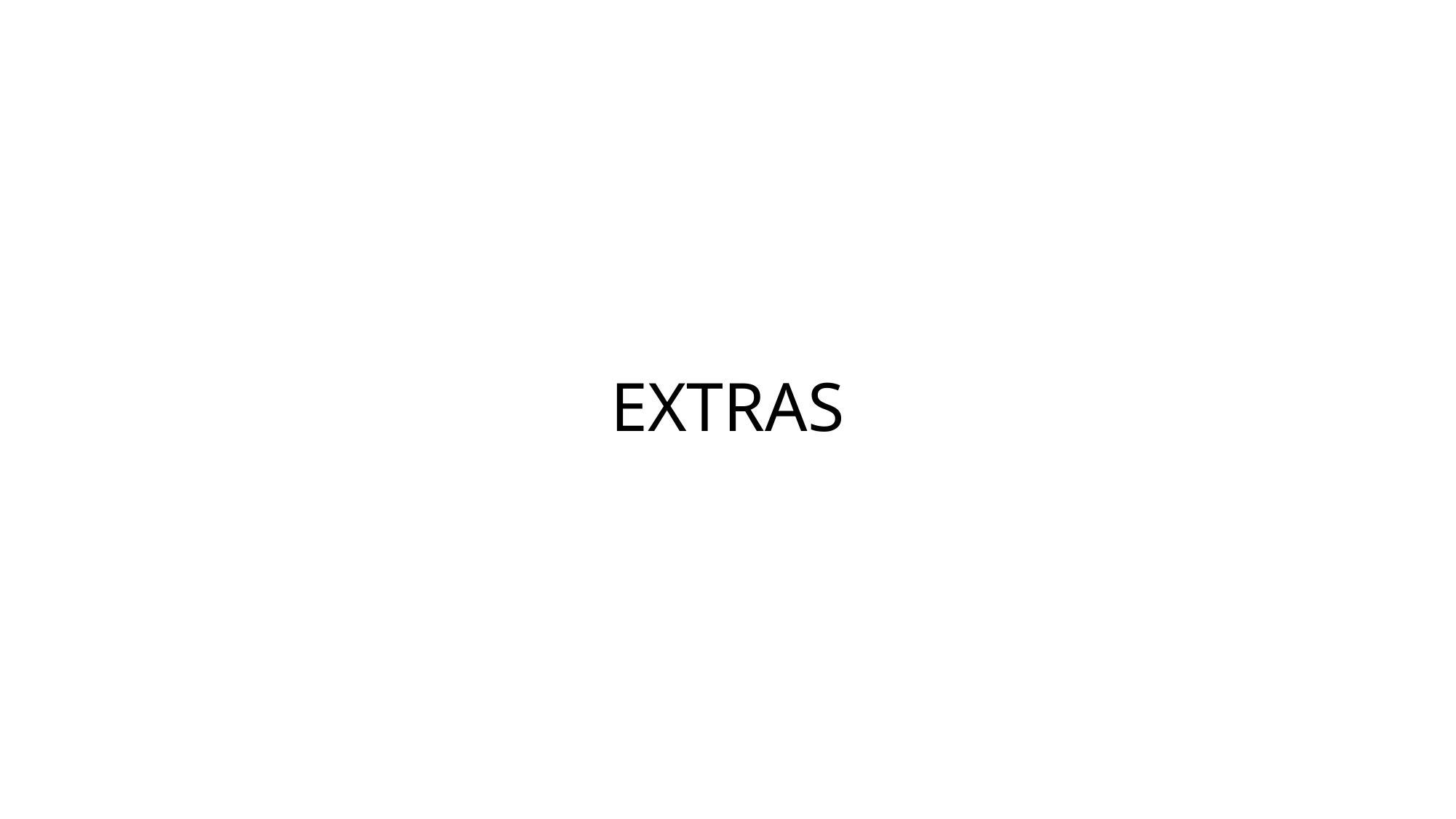

# EXTRAS

## Slide 15
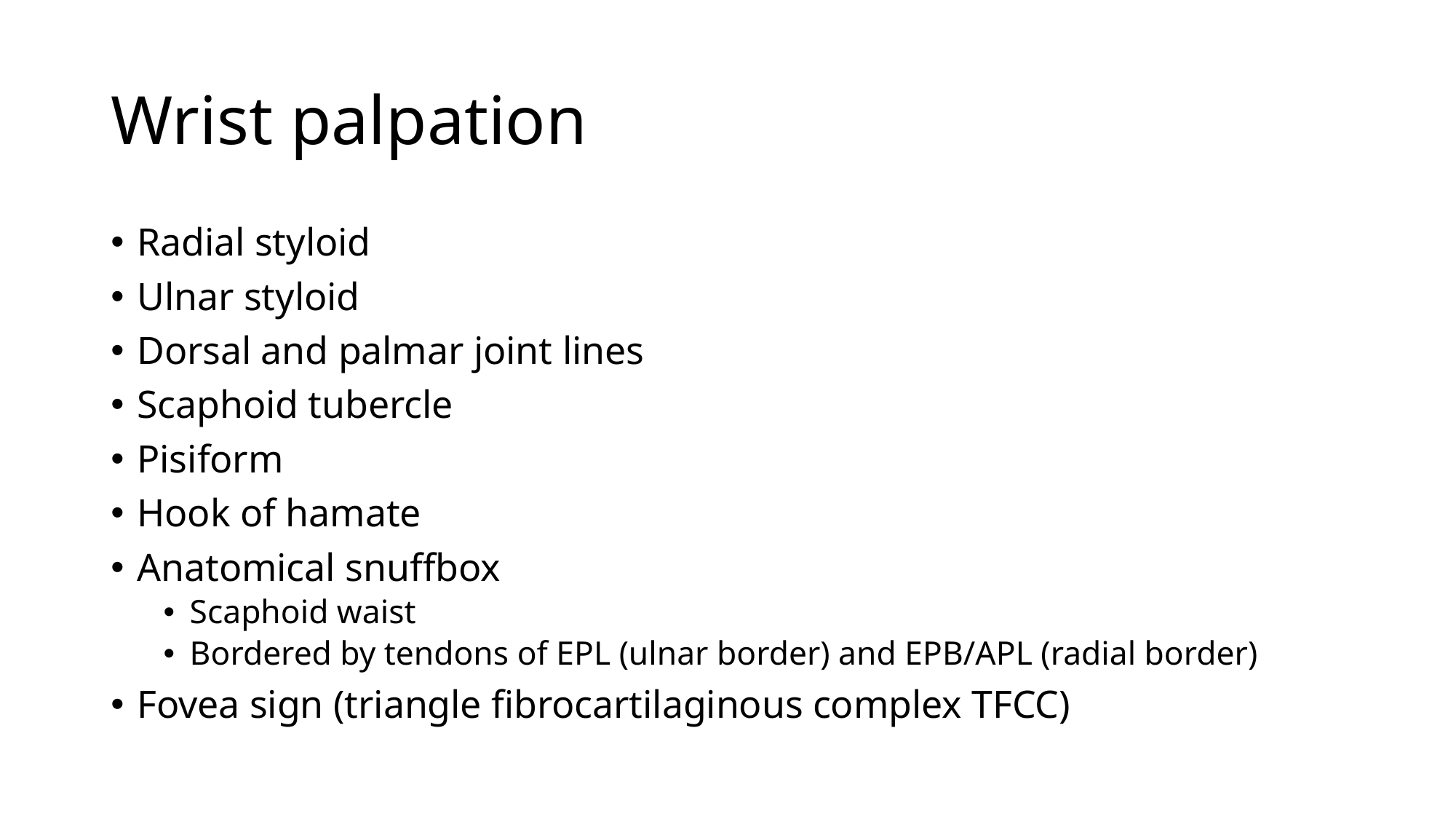

# Wrist palpation
Radial styloid
Ulnar styloid
Dorsal and palmar joint lines
Scaphoid tubercle
Pisiform
Hook of hamate
Anatomical snuffbox
Scaphoid waist
Bordered by tendons of EPL (ulnar border) and EPB/APL (radial border)
Fovea sign (triangle fibrocartilaginous complex TFCC)

## Slide 16
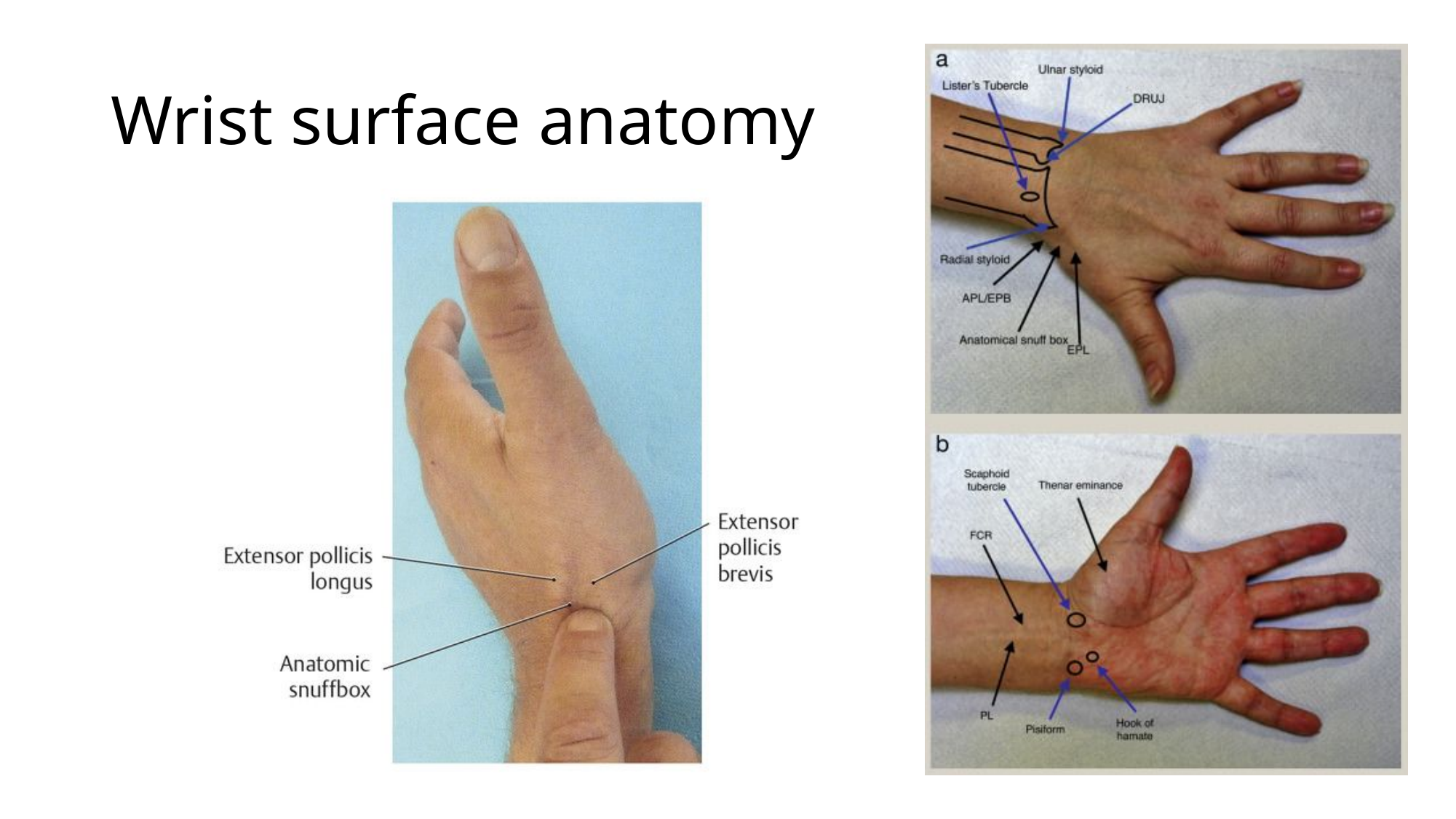

# Wrist surface anatomy

## Slide 17
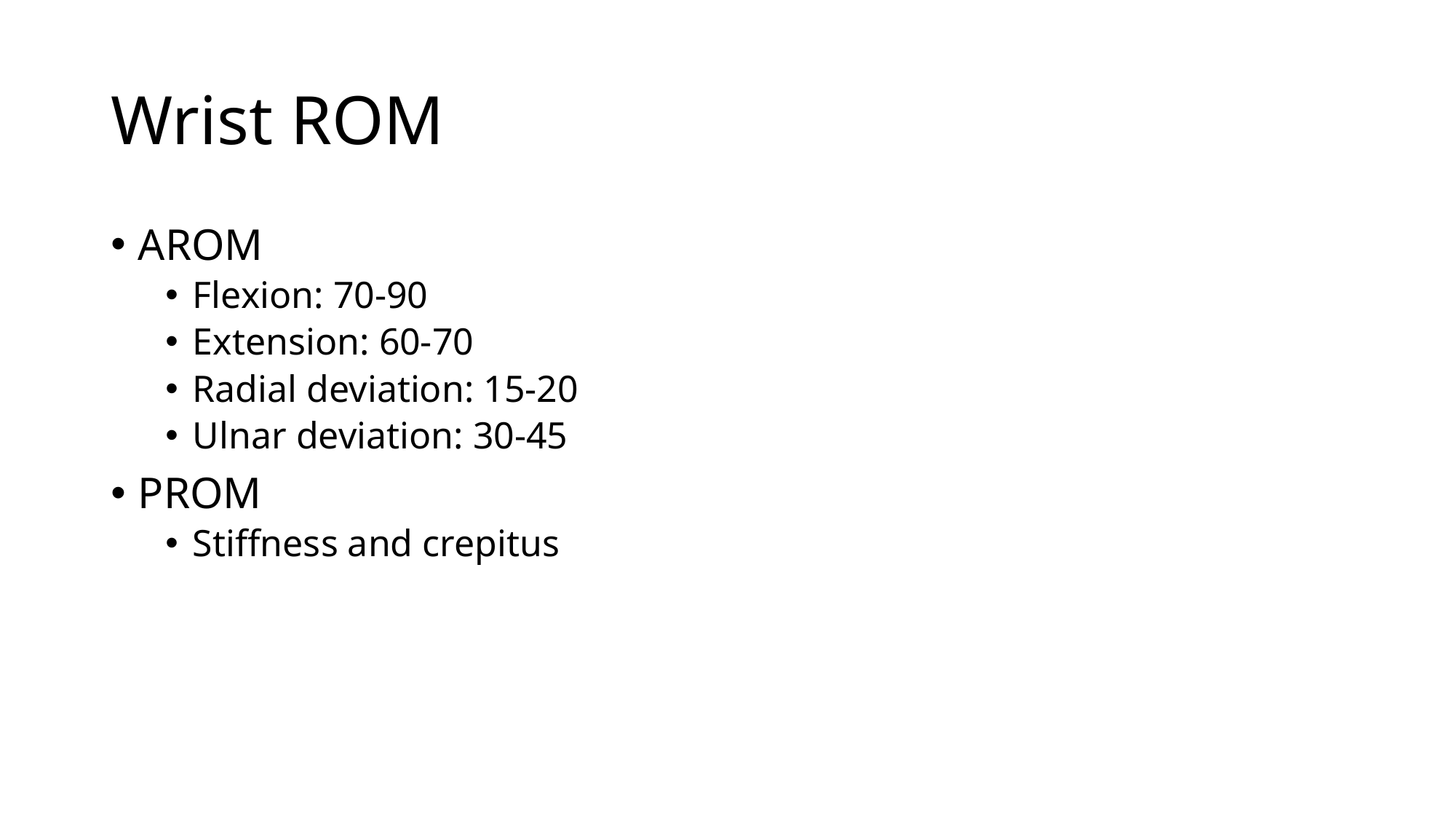

# Wrist ROM
AROM
Flexion: 70-90
Extension: 60-70
Radial deviation: 15-20
Ulnar deviation: 30-45
PROM
Stiffness and crepitus

## Slide 18
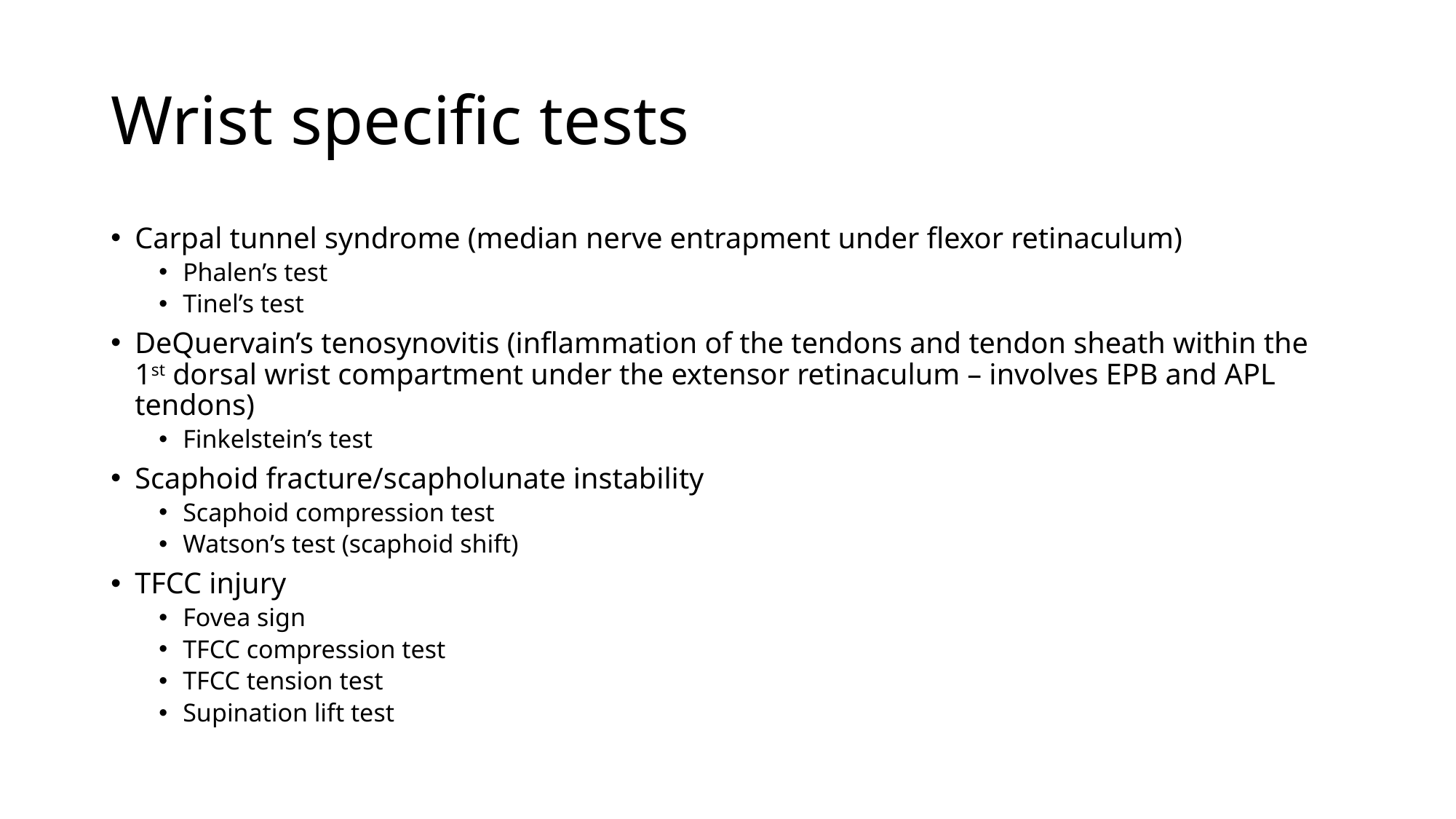

# Wrist specific tests
Carpal tunnel syndrome (median nerve entrapment under flexor retinaculum)
Phalen’s test
Tinel’s test
DeQuervain’s tenosynovitis (inflammation of the tendons and tendon sheath within the 1st dorsal wrist compartment under the extensor retinaculum – involves EPB and APL tendons)
Finkelstein’s test
Scaphoid fracture/scapholunate instability
Scaphoid compression test
Watson’s test (scaphoid shift)
TFCC injury
Fovea sign
TFCC compression test
TFCC tension test
Supination lift test
