# Supplementary material for: Enhancing musculoskeletal examination skills through near-peer teaching: student outcomes and perspectives
Source: BMC Med Educ. 2026 Apr 18;26:1012. doi: 10.1186/s12909-026-09235-2 (PMC13285311; doi:10.1186/s12909-026-09235-2)
Supplement: Supplementary file 3 — Supplementary Material 3. [file 12909_2026_9235_MOESM3_ESM.docx]

**Phase 1: Physical Examination – Musculoskeletal: Upper Limb**

**Student Name:** _______________________________________

**Student Number:** ____________________

**Campus:** ______

| **Assessment items** | ***Quality of performance** | | | | | |
| --- | --- | --- | --- | --- | --- | --- |
|  | **U** | **MU** | **MS** | **CS** | **E** | **Comments** |
| 1. General inspection:   □ Postural asymmetry  □ Scars, rashes  □ Swelling at other joints  □ Asks about pain | □ | □ | □ | □ | □ |  |
| 1. Limb inspection from back, front and side:   □ Posture  □ Asymmetry  □ Wasting  □ Swelling  □ Erythema  □ Scars, rashes | □ | □ | □ | □ | □ |  |
| 1. Palpation: elbow   □ Warmth  □ Swelling  □ Tenderness: lateral and medial epicondyles, olecranon | □ | □ | □ | □ | □ |  |
| 1. Range of movement: elbow   □ Flexion/extension  □ Supination/pronation  □ Passive range of movement | □ | □ | □ | □ | □ |  |
| 1. Special tests: elbow   □ Assessment for tennis elbow (palpation, resisted wrist extension) | □ | □ | □ | □ | □ |  |
| 1. Palpation: shoulder   □ Landmarks: sternoclavicular, acromioclavicular joints and the humeral head  □ Swelling  □ Tenderness  □ Warmth | □ | □ | □ | □ | □ |  |
| 1. Shoulder: active range of movement   □ Abduction/adduction  □ Flexion/extension  □ External rotation  □ Internal rotation | □ | □ | □ | □ | □ |  |
| 1. Shoulder: Passive range of movement   □ Compare both sides  □ Abduction/adduction  □ Flexion/extension  □ External rotation  □ Internal rotation |  |  |  |  |  |  |
| 1. Stability: shoulder   □ Sulcus sign (inferior instability)  □ Apprehension test (anterior instability) | □ | □ | □ | □ | □ |  |
| 1. Special tests: shoulder   □ Hawkins-Kennedy test (impingement test)  □ Painful arc (assessed during abduction) | □ | □ | □ | □ | □ |  |
| 1. Symmetry – check for equivalence right to left for all domains | □ | □ | □ | □ | □ |  |
| 1. Performs examination without causing pain, distress or embarrassment | □ | □ | □ | □ | □ |  |
| 1. Performs organised and efficient examination of the system within the nominated timeframe | □ | □ | □ | □ | □ |  |
| 1. Infection control:   □ Hand hygiene at beginning  □ Appropriate dress, nails, jewellery  □ Cleans equipment before and after use  □ Hand Hygiene at the end | □ | □ | □ | □ | □ |  |
| 1. Communication:   □ Introduces themselves and explains what they will do  □ Obtains permission to conduct the exam  □ Gives appropriate instructions and explanations to the patient throughout  □ At the end, thanks the patient for allowing them to conduct the examination | □ | □ | □ | □ | □ |  |

**General comments to student**

__________________________________________________________________________________

__________________________________________________________________________________

__________________________________________________________________________________
